# A 3D primary human cell-based in vitro model of non-alcoholic steatohepatitis for efficacy testing of clinical drug candidates

**DOI:** 10.1038/s41598-021-01951-7

**Published:** 2021-11-23

**Authors:** Simon Ströbel, Radina Kostadinova, Katia Fiaschetti-Egli, Jana Rupp, Manuela Bieri, Agnieszka Pawlowska, Donna Busler, Thomas Hofstetter, Katarzyna Sanchez, Sue Grepper, Eva Thoma

**Affiliations:** InSphero AG, Wagistrasse 27A, 8952 Schlieren, CH Switzerland

**Keywords:** Drug screening, Drug discovery, Diseases, Disease model

## Abstract

Non-alcoholic steatohepatitis (NASH) is a progressive and severe liver disease, characterized by lipid accumulation, inflammation, and downstream fibrosis. Despite its increasing prevalence, there is no approved treatment yet available for patients. This has been at least partially due to the lack of predictive preclinical models for studying this complex disease. Here, we present a 3D in vitro microtissue model that uses spheroidal, scaffold free co-culture of primary human hepatocytes, Kupffer cells, liver endothelial cells and hepatic stellate cells. Upon exposure to defined and clinically relevant lipotoxic and inflammatory stimuli, these microtissues develop key pathophysiological features of NASH within 10 days, including an increase of intracellular triglyceride content and lipids, and release of pro-inflammatory cytokines. Furthermore, fibrosis was evident through release of procollagen type I, and increased deposition of extracellular collagen fibers. Whole transcriptome analysis revealed changes in the regulation of pathways associated with NASH, such as lipid metabolism, inflammation and collagen processing. Importantly, treatment with anti-NASH drug candidates (Selonsertib and Firsocostat) decreased the measured specific disease parameter, in accordance with clinical observations. These drug treatments also significantly changed the gene expression patterns of the microtissues, thus providing mechanisms of action and revealing therapeutic potential. In summary, this human NASH model represents a promising drug discovery tool for understanding the underlying complex mechanisms in NASH, evaluating efficacy of anti-NASH drug candidates and identifying new approaches for therapeutic interventions.

## Introduction

Non-alcoholic fatty liver disease (NAFLD) defines a spectrum of pathologies, ranging from simple steatosis to its most severe form: non-alcoholic steatohepatitis (NASH), where it is believed that lipid accumulation in the liver (steatosis) and gut derived endotoxins results in inflammatory and fibrotic processes^1–3^. Liver fibrosis is defined as an excessive amount of fibrillar extracellular matrix (ECM) proteins, e.g. collagen I and III, within the space of Disse^2^. While initial matrix deposition is cleared in healthy conditions, deposition and clearing eventually get out of equilibrium in NASH, leading to blockage of blood flow along the liver acinus. This may ultimately lead to hepatocellular cancer (HCC) and/or cirrhosis, whose only option is liver transplant^3^. Different epidemiological studies estimate that more than 20% of the adult world population is already currently affected by some stage of NAFLD^4–6^. Extensive research has revealed that initiation and progression of this disease often takes decades. Contributors and mechanisms of disease progression are diverse and range from environmental factors such as over-nutrition and sedentary lifestyle^7,8^ to genetic predispositions, such as SNP variations in single genes^5^. Therefore, the identification of effective drugs has been an extremely complex process. To date, no high-throughput preclinical human model which recapitulates the different hallmarks of NASH has been available to researchers. In vivo animal models have been historically utilized for NASH pre-clinical drug discovery and research^7,8^. While there are some obvious benefits with in vivo animal models, such as the systemic components of NASH, there are also major disadvantages. Such shortcomings include species differences in metabolism and nutrition^9^, differences in regulation of NASH-linked genes such as HSD17β13^10^, and the need for strong stimuli such as hepatotoxicants (i.e. carbon tetrachloride) to induce liver fibrosis in the short lifespan of the animal^1^. Furthermore, animal models of NASH which are induced by high fat/high carbohydrate diet can recapitulate steatosis, inflammation and perhaps initial fibrosis F1 and F2, but not advanced stages F3 and F4^11^. Therefore, these models are not ideal for efficacy assessment of anti-fibrotic compounds. More relevant and predictive human models are needed, not only to better understand NASH and its underlying mechanisms, but also to evaluate potential anti-NASH drug candidates.

However, it has been challenging for existing in vitro human models to predict the in vivo effects of drugs. NASH involves a complex interplay between different organs^12^ and cell types^13^, all of which need to be taken into account when developing an in vitro model. The three major hallmarks of NASH involve multiple liver cell types, including hepatocytes and non-parenchymal cells (NPCs); specifically: tissue resident Kupffer cells (KCs), liver endothelial cells (LECs) and hepatic stellate cells (HSCs)^14^. Following initial hepatocellular accumulation of lipids (triglycerides, TGs)^5,6^, KCs are the first macrophages to be activated in NAFLD, and thus are of critical importance in the progression of NAFLD to NASH. KCs, as well as hepatocytes and LECs, release cytokines e.g. TGF-β, PDGF-α/β, VEGF-α, etc.^15,16^ that promote the activation of HSCs into myofibroblasts^2^. Myofibroblasts deposit fibrosis-associated proteins, including collagen I, III and fibronectin, resulting in scar formation and ultimately cirrhosis^1,17,18^. Therefore, in order to recapitulate steatosis, inflammation and fibrosis, all of these cells must be included in an in vitro NASH research model.

Regardless, in recent years remarkable progress has been made in the development of human cell-based in vitro models for NASH and NAFLD. Basic models employing 2D monolayer cultures of single cell types can be used to mimic part of the NASH pathogenesis (e.g. steatosis) and model the lipid-reducing effects of anti-steatotic compounds^19,20^. More advanced cell culture technologies include 3D culture, microfluidics or a combination of both. For example, Prill^21^ developed a 3D model that enables the long-term culture of primary human hepatocytes (PHHs) and induction of lipid loading upon exposure to defined stimuli. While these 2D and 3D monoculture approaches allow a recapitulation of the early stages of NAFLD i.e. lipid metabolism, they cannot be used to investigate compound effects on downstream events in inflammation and fibrosis, since they lack all the relevant cells involved^14^. To address this, another elegant approach was developed which incorporated PHHs, KCs, and HSCs. In this model, the PHHs were cultured in a transwell format under flow conditions, whereby inflammatory and fibrotic pathways were activated using lipotoxic stimuli^22^. However, since the cells were cultured in a 2D format, with PHHs physically separated from NPCs, this model lacked adequate crosstalk between cells.

Another aspect to consider for predictive model development is the use of NASH-relevant stimuli. Free fatty acids (FFA) play a major role in NASH initiation. Unsaturated oleic acid and saturated palmitic acid are the two most abundant fatty acids in Western diets, and both are involved in the development of NASH^23^. Sugars are also cited as critical drivers for NAFLD initiation and progression. This includes not only glucose but also other sugars^24^ which needs to be appropriately represented in models of NAFLD. Lastly, inflammatory stimuli are critical for NAFLD progression, and are often considered the “second hit” of disease progression. In NASH patients, increased levels of circulating lipopolysaccharide (LPS) are thought to originate from disturbed gut microbiome^25^, which stimulate hepatocytes and KCs to trigger inflammation and profibrotic processes^1,25^.

Therefore, we have developed a complex 3D human in vitro model, which incorporates all relevant liver cell types, as well as clinically relevant NASH stimuli necessary to recapitulate the main pathophysiological hallmarks of this disease. The human liver microtissues (hLiMTs) contain PHHs, LECs, KCs and HSCs at a physiologically relevant ratio^26–28^. Since in this model only liver tissue residential and no peripheral mononucleated cells (i.e. macrophages), responsible for resolution of fibrosis, were incorporated, the prevention of NASH onset and not the reversion of the disease was investigated. The combination of a 96-well plate format with scalable, quantifiable endpoints enables the high-throughput assessment of drug candidates. The studies presented here describe the characterization of this 3D NASH model. Furthermore, extensive model validation was performed using tool compounds and clinical NASH candidates targeting every relevant disease aspect (i.e. steatosis, inflammation and/or fibrosis).

For example, effects on the anti-steatotic and anti-fibrotic aspects of NASH were investigated using an acetyl-CoA carboxylase (ACC) inhibitor Firsocostat. ACC catalyzes the rate-limiting step of de novo lipogenesis and is involved in the regulation of mitochondrial fatty acid oxidation. Increased levels of lipogenesis and reduced fatty acid oxidation are thought to contribute to steatosis. The inhibition of ACC and de novo lipogenesis has been shown to repress the activation of HSCs^29,30^. Indeed, this clinical drug candidate Firsocostat has been shown to reduce hepatic fat and markers of liver injury in patients with NASH^6,31,32^, making it an excellent compound to test in our 3D model.

In addition to Firsocostat, direct anti-inflammatory and anti-fibrotic compounds were validated as well, including the apoptosis signal-regulating kinase 1 (ASK1) inhibitor, Selonsertib. ASK1 is a serine/threonine kinase activated by oxidative stress, resulting in inflammation and apoptosis subsequently leading to liver fibrogenesis^33^. In a similar in vitro NASH model to the one presented here, Selonsertib was shown to decrease both, HSC activation and pro-inflammatory responses, thereby reversing fibrosis induction^23^.

Since fibrosis is considered to be the most detrimental stage of NASH, it was imperative to validate our model with compounds that effect these fibrotic pathways. TGF-β is a major pro-fibrotic mediator released by several liver cell types. TGF-β activates quiescent HSCs to a myofibroblast phenotype producing fibrillary collagen^18^. In studies described here, an anti-fibrotic tool compound activin receptor-like kinase 5 inhibitor (ALK5i) was tested for efficacy. ALK5i has been previously shown to inhibit TGF-β1 signaling in fibrotic precision cut liver slices (PCLS) and prevent collagen production/matrix deposition from activated thereby limiting fibrogenesis^34,35^.

We demonstrate that the prolonged co-culture of PHHs, KCs, LECs and HSCs, exposed to FFAs and LPS, allows longitudinal changes that approximate disease progression and mimic the observed features of NASH. The in vitro 3D NASH model, described here, is well-suited as a tool for drug discovery, to be used either to study the molecular mechanisms of the disease or for the screening of anti-NASH compounds.

## Materials and methods

### Liver microtissue maintenance and disease induction

3D InSight hLiMTs were composed of a pool of PHHs from 10 donors, single-donor HSCs and single-donor KCs/LECs (MT-02-302-05, InSphero AG). hLiMTs were generated by self-assembly of monodispersed primary cells, as described previously^26–28^. hLiMTs were maintained at 37 °C, 5% CO_2_, 95% humidity.

**Figure 1:**
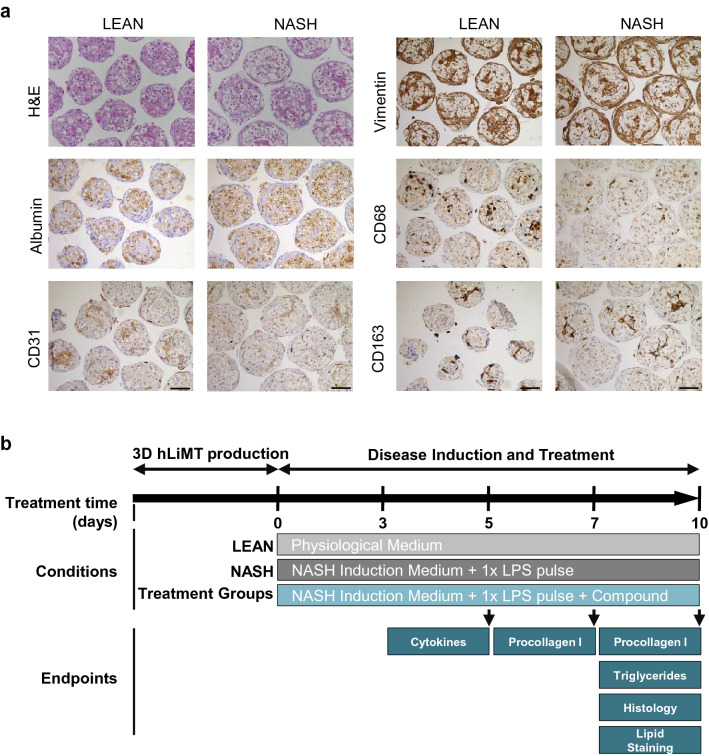
Method and characterization of hLiMTs for modeling NASH (**a**) H&E and IHC staining of liver specific makers representing the relevant cell types in the liver model. Scale bars indicate 100 μm. (**b**) Scheme for NASH induction. Disease induction and treatment starts at day 0 after the production of the 3D InSight hLiMTs. The three treatment conditions are: LEAN, NASH and NASH in the presence of test compound. After each medium exchange on days 0, 3, 5 and 7, the compounds and DMSO were applied. FFA supplementation occurred on each treatment day, while stimulation with LPS took place only once during the 10-day treatment period. Cell culture supernatants and/or microtissues were collected on days 5, 7 and 10. All conditions were normalized to 0.2% DMSO.

To mimic NASH disease induction and progression, hLiMTs were exposed for 10 days to either LEAN or NASH conditions (Fig. 1b). LEAN condition hLiMTs were cultured in basal hepatocyte maintenance medium (CS-07-302-01, InSphero AG). Under NASH conditions, the medium was supplemented with elevated glucose and fructose levels (total of 22.5 mM) (CS-07-301-01, InSphero AG), free fatty acids (FFAs, 167 µM, CP-02-302, CP-02-303, InSphero AG), and lipopolysaccharide (5 µg/ml LPS, Sigma-Aldrich). LPS was applied as a pulsing at day 3 of treatment. Medium was exchanged on days 0, 3, 5, and 7.

### Compound treatment

Firsocostat (HY-16901, MedChemExpress), Selonsertib (HY-18938, MedChemExpress) or ALK5i, (SB525334, Selleckchem), were applied during the 10-day NASH induction protocol. Stock solutions were prepared in DMSO. Final concentrations: 0.5 µM and 10 µM Firsocostat, 0.5 µM, 2 µM and 10 µM Selonsertib and 0.1 µM, 0.5 µM and 2 µM ALK5i. Compound treatments were performed on days 0, 3, 5 and 7, using a TECAN D300e Digital Dispenser and all conditions were normalized to 0.2% DMSO.

### Endpoint assays

All assays were performed with 4–6 biological replicates per treatment group and were repeated at least twice. For all experiments the same primary human hepatocyte (PHH) cell lot was used. The PHH cell lot consisted of a mixture of ten different donors.

#### LDH release

Activity of lactate dehydrogenase (LDH) released from damaged cells into the supernatant was measured using the LDH-Glo Cytotoxicity Assay kit (Promega Corporation), according to manufacturer’s protocol. Cell culture supernatants were diluted 1:5 in LDH storage buffer and the diluted samples (25 µl) were mixed with 25 µl detection reagent in white half area assay plates (Greiner Bio-One). After a 1 h incubation on an orbital shaker at room temperature (RT), protected from light, luminescence was measured with a Tecan Spark 10 M plate reader. Measured relative luminescence unit values (RLUs) are directly proportional to the LDH concentration. Background-subtracted RLU values are plotted as mean ± SD, including individual values. Calculations were done in Microsoft Excel and graphs prepared with GraphPad Prism (Version 8) software.

#### Triglyceride assay

Intracellular TG content was measured with Promega’s Triglyceride-Glo assay (Promega Corporation). hLiMTs were washed with PBS (Sigma-Aldrich) and lysed in lysis solution (25 µl, including lipase) for 40 min on an orbital shaker. The lysates were vigorously mixed before transfer to white half area assay plates (Greiner Bio-One) where detection reagent (25 µl) was added. After a 1 h incubation on an orbital shaker at RT, protected from light, luminescence was measured with a Tecan Spark 10 M plate reader. Free glycerol background in hLiMTs were negligible. The TG amount per hLiMT was calculated by linear fit to a glycerol standard curve prepared in lysis solution. Values were plotted as mean ± SD, including individual values, with GraphPad Prism (Version 8). Either unpaired, two-tailed t-test with Welch’s correction vs NASH or the Welch and Brown-Forsythe version of one-way ANOVA in combination with Dunnett’s T3 multiple comparisons test vs. NASH was used to calculate significant changes in TG content, *p < 0.05.

#### Procollagen I measurement

The levels of cleaved and secreted C-terminal human collagen type I pro-peptide in hLiMT supernatants collected on days 7 and 10 were measured with the human procollagen I homogeneous time-resolved fluorescence (HTRF) assay (Cisbio). Undiluted samples were processed according to the supplier’s instructions. Fluorescence was measured with a Tecan Spark 10 M plate reader. Procollagen I amount released per hLiMT was calculated by linear fit to a standard curve. Values normalized to incubation time were plotted as mean ± SD, including individual datapoints. Unpaired, two-tailed t-test with Welch’s correction vs NASH or the Welch and Brown-Forsythe version of one-way ANOVA in combination with Dunnett’s T3 multiple comparisons test vs. NASH was used to calculate significant changes, *p < 0.05.

#### Cytokine secretion

Cytokines (IL-6, TNF-α, MIP-1α (CCL3), MCP-1, IP-10 (CXCL10), IL-8 (CXCL8)) in cell supernatants were determined on day 5 using the Magnetic Luminex Performance Assay (Human XL Cytokine Discovery Base Kit, LUXLM000, beads: LUXLM206, LUXLM208, LUXLM266, LUXLM279, LUXLM210, LUXLM270, Bio-Techne). The multiplexed assay was performed according to supplier’s instructions with minor adaptation of microparticle and antibody concentrations to analyte levels present in supernatants. Measurements were performed with a Luminex MAGPIX analyzer. Protein concentrations were calculated by five parameter logistic fit to a standard curve using Luminex XPonent software. Data are represented as mean values ± SD, including individual datapoints. An unpaired, two-tailed t-test with Welch’s correction vs NASH or the Welch and Brown-Forsythe version of one-way ANOVA in combination with Dunnett’s T3 multiple comparisons test vs. NASH was used to calculate significant changes, *p < 0.05. Outliers were detected based on Grubbs’ test (α = 0.05).

#### Formalin-fixation and paraffin-embedding (FFPE) of hLiMTs

On day 10 of treatment, 18–24 hLiMTs per treatment group were pooled, washed with PBS and fixed with 4% paraformaldehyde (PFA, Alfa Aesar) for 1 h at RT. The fixed hLiMTs were washed in PBS, pelleted in 1.7% agarose, and further processed with a tissue processor (Logos Microwave Hybrid Tissue Processor; Milestone Medical S.r.I) for paraffin embedding. The paraffin blocks were sectioned to 4 μm thickness using a microtome (Histocore Multicut, semimotorized Rotation Microtome; Leica Biosystems). The sections were mounted on poly-L lysin treated glass slides (HistoBond + S with grounded edge; Marienfeld) and dried for 60 min at 65 °C.

#### Immunohistological and histochemical staining (IHC and HC)

To demonstrate the presence of relevant liver cell types in the LEAN and NASH hLiMT, standard immunohistochemical staining methods were performed with primary and secondary antibody labelled with HRP (rabbit anti-mouse secondary antibody and a goat anti-mouse compact polymer labelled with HRP; polyclonal rabbit anti-goat, Abcam, dilution 1:500). HRP was detected using DAB, followed by counterstain with hematoxylin. The following antibodies were used: CD31 (Clone JC70; Cell Marque, dilution 1:200); CD68 (monoclonal mouse anti-human CD68; Clone 514H12, Novocastra, dilution 1:150); CD163 (monoclonal mouse anti-human CD163; Clone EDHu-1, Serotec, dilution 1:1000); Coll I (monoclonal mouse anti-human collagen I; Clone 3G3, Abcam, dilution 1:250); Coll III (polyclonal rabbit anti-human collagen III, Abcam, dilution 1:200; pre-treatment); Albumin (polyclonal goat anti-human albumin, Bethyl Lab, dilution 1:5000) and Vimentin (monoclonal mouse anti-human vimentin; Clone EPR3776, Abcam, dilution 1:3000). Hematoxylin (Mayers Hematoxylin, Leica Biosystems) and eosin (eosin 2% aqueous, Chroma Waldeck) staining was performed as described previously^36^. For visualization of fibrillated collagen structures, a Sirius Red (SR) staining procedure, according to Kiernan^37^ with slight modifications, was applied. After rehydration, the sections were stained with Sirius Red solution (Sirius Red solution, Chroma Waldeck) for 5 min and stopped with 0.5% acidic acid solution (without hematoxylin counterstaining). Histological sections images were captured using a Leica DMi8 microscope at 10× (HC PL FLUOTAR 10×/0.32 PH1) or 20× (HC PL FL L 20×/0.40 CORR PH1) magnification. Automated white balance correction was applied, and images were captured with a DMC4500 digital camera. For the polarized light images, a motorized, polarized filter was used. Images were inverted using ImageJ invert function for better visualization.

#### Nile red staining

On day 10 of treatment, hLiMTs were washed once with PBS (Sigma-Aldrich) and fixed for 1 h at RT in 4% PFA (Alfa Aesar). PBS was removed and staining solution PBS + Nile Red (1:700 from 1 mg/ml stock in acetone, Sigma-Aldrich) + DAPI (1:500 from 1 mg/ml stock solution in ddH_2_O, Sigma-Aldrich)] was added. After 1 h incubation at RT, the staining solution was removed and hLiMTs were washed once with PBS before imaging.

#### High content image acquisition and analysis

Imaging was performed on a Yokogawa CQ1 confocal high content analysis instrument (Yokogawa Electric Corp., Tokyo, Japan) with a 40× objective (LUCPLFLN40X, Olympus). Settings applied for the different channels were: DAPI: excitation 405 nm, laser power 30%, emission filter 447/60; Nile Red: excitation 488 nm, laser power 20%, emission filter BP617/73. Image stacks with ~ 4 µm distance between image layers were acquired, with a total depth of 62 µm and 15 sections per hLiMT. For image analysis, maximum intensity projections (MIPs) were created from z-sections for both channels. The DAPI channel was used to identify the entire hLiMT region. Wells without any hLiMTs detected were excluded from the analysis. Lipid areas were identified based on Nile Red channel. Only lipid areas within the hLiMT region were analyzed. Relative lipid area was calculated as a percentage for each individual hLiMT using the formula: (Relative Lipid Area) = (number of lipid area objects)*(average lipid area per lipid area object)*100/(hLiMT area). Unpaired, two-tailed t-test with Welch’s correction vs NASH or the Welch and Brown-Forsythe version of one-way ANOVA in combination with Dunnett’s T3 multiple comparisons test vs. NASH was used to calculate significant changes, *p < 0.05. Representative images are shown as MIPs.

#### RNA sequencing and analysis

RNA Sequencing (RNA-Seq) was performed by BioSpyder Technologies, Inc. using TempO-Seq technology^38^. The Human Whole Transcriptome 2.0 assay, consisting of 22537 probes targeting 19701 genes, was utilized. Sample generation process was as follows: on days 5 and 10 of treatment, single hLiMTs were washed in PBS without Ca^2+^/Mg^2+^ and lysed in 15 μl of 1× Enhanced Lysis Buffer (BioSpyder Technologies, Inc.). Lysates were sent to BioSpyder for library generation and sequencing using the Illumina sequencing platform. After sample demultiplexing and obtaining single FASTQ files, reads alignment and counting were performed using TempO-SeqR data analysis platform (BioSpyder Technologies, Inc.). Further TempO-Seq data analysis was done by InSphero via proprietary R-based^39^ analytical pipeline. Probe-wise raw count table was collapsed toward gene-wise count table by summating counts for probes associated with the same gene. Low-expression genes (defined as genes for which number of samples with non-zero counts were less than 15% of all samples) were filtered out. Data normalization, Principal Component Analysis and Differential Expression Analysis (DEA) were performed as implemented in *DESeq2* R package^40^. Furthermore, Surrogate Variable Analysis (SVA)^41^ was applied to remove unintended batch effects by using *sva* R package. Hierarchical clustering was performed using correlation distance metric and ward.D2 linkage method. Pre-ranked Gene Set Enrichment Analysis (GSEA)^42^ was performed using the *cluster Profiler* R library^27,43^. DEA output was used as input for GSEA with genes ranked according to log2FC. Normalized Enrichment Score (NES) was used as an enrichment metric. GSEA was run on MsigDB database^44^ version 7.4 selecting KEGG^45^, Hallmark^44^, PID^46^, Reactome^47^ and WikiPathways^48^ sub-collections. For both, DEA and GSEA, Benjamini–Hochberg procedure for False Discovery Rate (FDR)^49^ was used to correct p values across contrasts, and FDR cut-off was set to 0.05.

## Results

### Characterization of LEAN and NASH conditioned hLiMTs

#### Confirmation of maintenance of key cell types in 3D liver microtissues over 10-day culture period

First, the morphology and presence of the relevant liver cell types for disease inductions were confirmed in NASH and compared with LEAN hLiMTs. Figure 1a shows that the NASH and LEAN hLiMTs contained the main liver cells types responsible for disease initiation and progression, namely, PHHs (albumin containing cells), LECs (detected using the cell type specific marker, CD31) and HSCs (detected using the cell type specific marker, vimentin). The presence of KCs was detected by staining for CD68 (a pro-inflammatory M1 macrophage marker) and CD163 (a pro-fibrotic M2 macrophage marker)^50^. In addition, hematoxylin and eosin (H&E) staining revealed visible accumulation of lipids in NASH hLiMTs compared to LEAN hLiMTs. The treatment regimen used to induce NASH was not toxic to the hLiMTs, which maintained viability without signs of necrosis over the entire 10-day culture period (Supplementary Fig. S1).

### NASH treatment results in higher intracellular lipid content and TG levels

**Figure 2:**
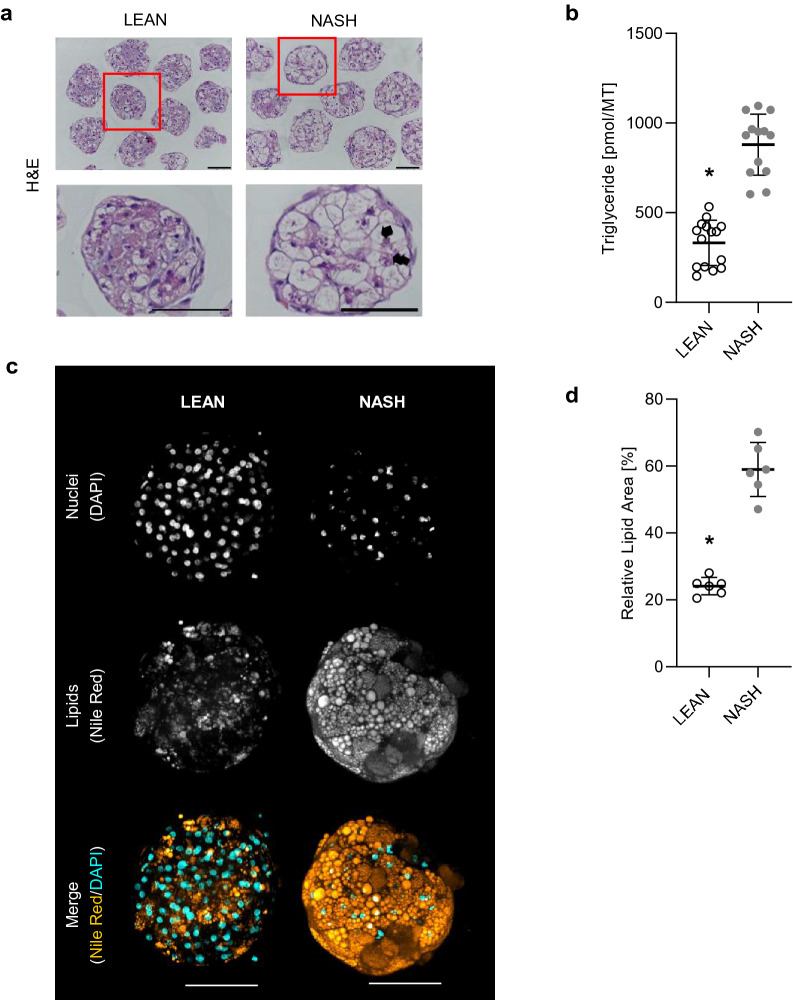
Steatosis-like phenotype and lipid accumulation in NASH-treated hLiMTs. (**a**) H&E staining demonstrating increased accumulation of lipids within the NASH hLiMTs compared to LEAN control hLiMTs. Ballooning of hepatocytes with displaced nuclei (arrows). Scale bars indicate 100 μm. (**b**) A significant increase (*p < 0.05) of tissue TG levels in NASH vs. LEAN control-treated hLiMTs, confirming the steatosis-like phenotype. TG data is a mean of three independent experiments (LEAN n = 15, NASH n = 13). (**c**) Intracellular lipid accumulation was assessed by Nile Red whole-mount staining and represented as maximal intensity projections. Increased intracellular lipid accumulation, as well as increased micro-vesicular (small lipid droplets) and macro-vesicular (big lipids droplets) steatosis was detectable in NASH vs. LEAN control hLiMTs. Scale bars indicate 100 μm. (**d**) High content image-based quantification of lipid content demonstrated significant increase of lipids in NASH treated hLiMTs compared to control hLiMTs (*p < 0.05).

Figure 2 shows the steatosis-like phenotype and lipid accumulation in NASH treated hLiMTs. In addition to the increased accumulation of lipids in NASH compared to LEAN hLiMTs, there was evidence of ballooning of hepatocytes with displaced nuclei (Fig. 2a). NASH hLiMTs also contained significantly higher levels of TG compared to LEAN hLiMTs (Fig. 2b), confirming the steatosis-like phenotype. High content imaging (HCI) of Nile Red stained microtissues demonstrated significant intracellular fat accumulation in NASH compared to LEAN hLiMTs (Fig. 2c). Confocal images further revealed the presence of micro-vesicular (small lipid droplets) and macro-vesicular (large lipids droplets) steatosis in NASH hLiMTs. In NASH treated hLiMTs the number of nuclei are less visible as compared to LEAN condition which can be explain by their displacement in ballooned hepatocytes under steatosis condition. Quantification of lipid droplets indicated a 2.5-fold increase of accumulated small and large lipid droplets in NASH compared to LEAN hLiMTs (Fig. 2d), which correlated well with the measured increase of TG in the NASH samples compared to LEAN hLiMTs.

### NASH treatment in 3D liver microtissues results in inflammation

**Figure 3:**
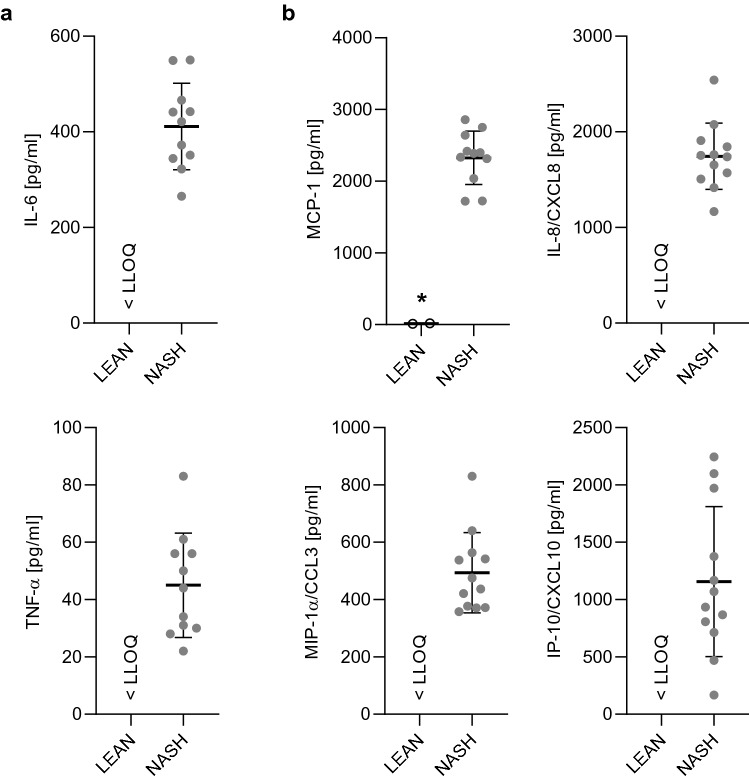
Induction of inflammation in NASH-treated hLiMTs. FFA and LPS-mediated induction of NASH leads to a pro-inflammatory phenotype compared to LEAN control, with the detection of (**a**) increased secretion of pro-inflammatory cytokines: IL-6 and TNF-α and (**b**) chemokines: MCP-1 (*p < 0.05), MIP-1α (CCL3), IL-8 (CXCL8) and IP-10 (CXCL10). Secreted cytokines/chemokines by various liver cells: KCs: IL-6, MIP-1α (CCL3), TNF-α, HSCs: IP-10 (CXCL10), MCP-1, IL-8, PHHS: IL-8, MCP-1 (3 experiments, n = 12). Lower level of quantification (LLOQ) was applied where quantification was below the lowest assay standard.

Cytokines and chemokines shown to be upregulated in serum of patients with NASH^51^ were also upregulated in NASH hLiMTs. NASH-treated microtissues have shown increased secretion of pro-inflammatory cytokines IL-6 and TNF-α (Fig. 3a) and pro-inflammatory chemokines MCP-1, MIP-1α, IL-8 and IP-10 (Fig. 3b). Conversely, the levels of these cytokines and chemokines were very low (MCP-1) or even below the lower limit of quantification (LLOQ) in LEAN hLiMTs. We assessed the cytokine and chemokines at day 5 due to their largest peak in secretion with sufficient sensitivity between LEAN and NASH conditions.

### Treatment with NASH-inducing stimuli leads to fibrotic phenotype in 3D liver microtissues

**Figure 4:**
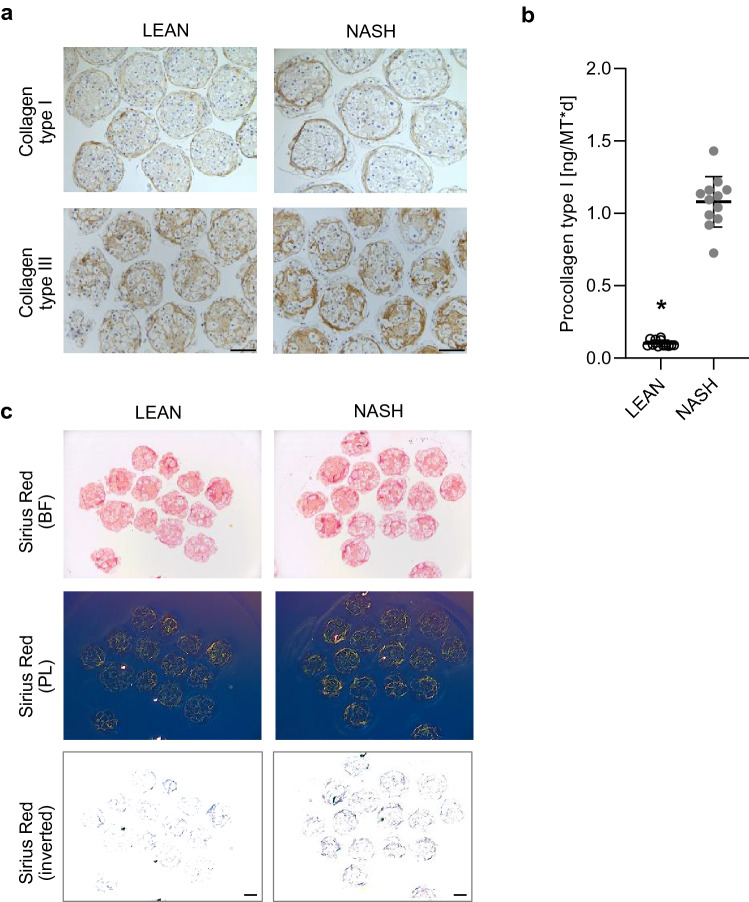
Induction of fibrosis in NASH-treated hLiMTs. (**a**) IHC analysis showed increased staining intensity of collagen type I and III in NASH vs. LEAN control hLiMTs. Scale bars indicate 100 μm. (**b**) Cleaved C-terminus pro-peptide of procollagen type I was measured in supernatants on day 7 and 10 and was normalized to secretion per hLiMT and per day. NASH-treated samples showed a significant increase (*p < 0.05) in procollagen I release compared to LEAN control hLiMTs (3 independent experiments, n = 12 for NASH and LEAN). (**c**) Sirius Red staining and polarized light microscopy of tissue sections showing collagen fibrillation. The results demonstrated an increased collagen fibrillation in NASH vs. LEAN control treated hLiMTs. Polarized light images are shown as inverted images for improved visualization. Scale bars indicate 100 μm.

To show the effect of NASH treatment on fibrosis development and HSC activation, the protein expression of collagen type I and III was investigated using IHC. The NASH hLiMTs expressed higher protein levels of both collagen type I and III than LEAN hLiMTs (Fig. 4a). Active collagen synthesis was evaluated by measuring the levels of cleaved C-terminal pro-peptide, a product of procollagen I synthesis, in the supernatants. The synthesis and secretion of procollagen type I was ~ 10-fold higher in NASH than LEAN hLiMTs (Fig. 4b), which correlated well with the level of collagen type I protein expression. Moreover, collagen fibrillation was visualized by Sirius Red staining of histological sections, a well-established method to qualify pathological changes of collagen fiber content and structure in clinical tissues sections^52^. We assessed tissue sections under bright and polarized light and showed an increased collagen deposition and fibrillation in NASH compared to LEAN hLiMTs (Fig. 4c).

### NASH treatment induces disease-relevant gene expression changes in human 3D liver microtissues

**Figure 5:**
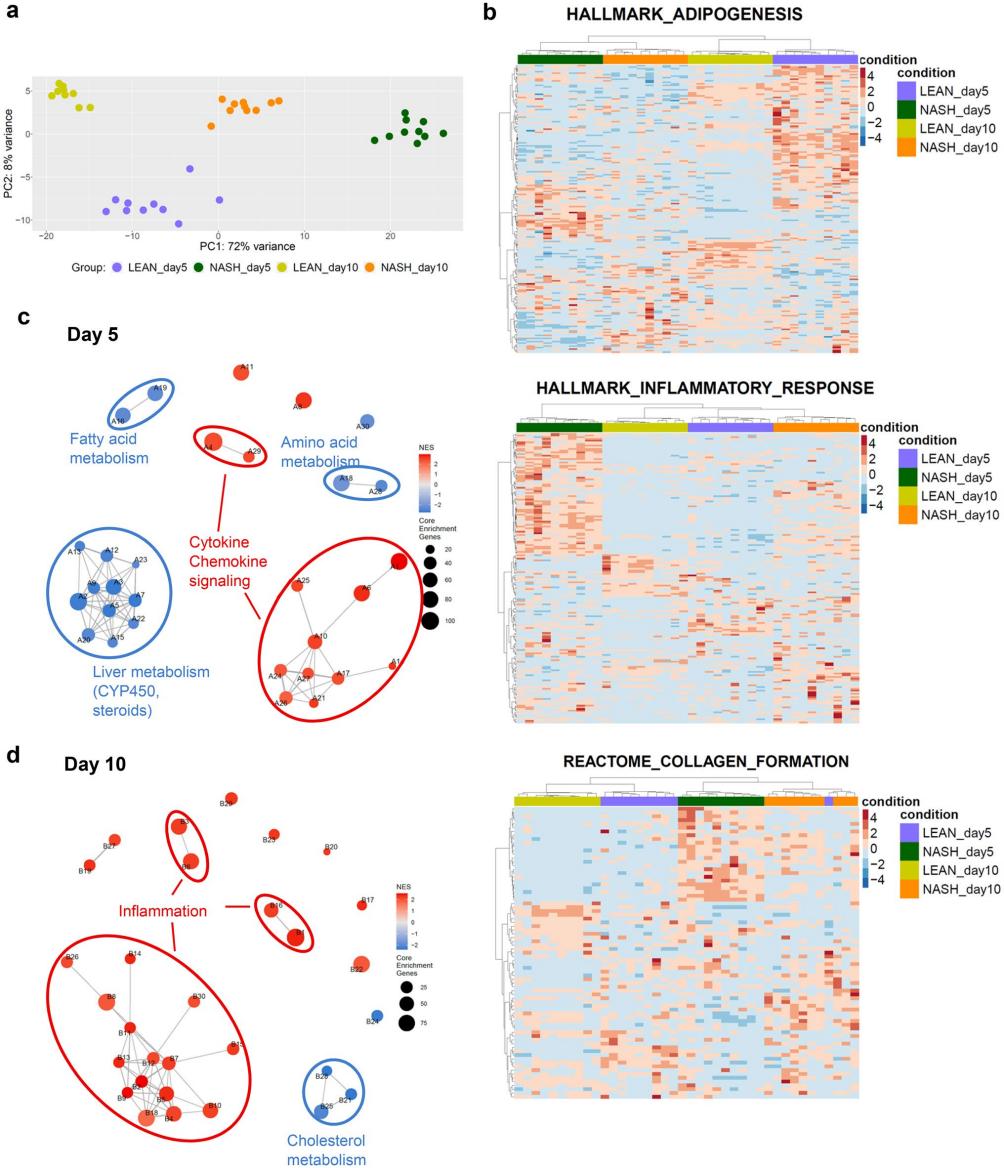
Global transcriptomic profiling of NASH hLiMTs confirms the induction of NASH phenotype. (**a**) Principal component analysis of whole transcriptome expression profiles from LEAN and NASH treatment groups on day 5 and 10. Distinct clustering of treatment groups suggest the robustness of the method. (**b**) Heatmaps for NASH and LEAN hLiMTs on day 5 and 10 for gene sets associated with hallmarks of NASH, namely adipogenesis (Hallmark, top panel), inflammatory response (Hallmark, middle panel) and collagen formation (Reactome, bottom panel). Columns represent samples, and rows are genes. Row-wise Z-score transformation was performed on log2 expression values for each gene, with blue denoting a lower and red a higher expression level according to the average expression level. Distinct expression patterns for NASH and LEAN hLiMTs achieved by unsupervised clustering indicate regulation of the indicated pathways upon NASH treatment. (**c**, **d**) Enrichment map of gene sets derived from GSEA comparing NASH to LEAN hLiMTs on (**c**) day 5 and (**d**) day 10 of treatment. The 30 most significantly regulated gene sets are displayed. Color scale represents normalized enrichment score (NES). Red nodes represent gene sets upregulated in NASH, whereas blue nodes represent gene sets downregulated. The sizes of the nodes depict the numbers of core enrichment genes. Nodes are grouped by their similarity, with connecting lines representing common genes between nodes. Cluster of functionally related nodes were summarized and annotated accordingly. Full gene set lists are provided in Supplementary Tables S1 and S2. Data points are derived from single hLiMTs (n = 10), results from one of two independent experiments are shown.

To characterize the NASH phenotype on a molecular level, we performed whole transcriptomic profiling on days 5 and 10 of NASH induction, using a targeted sequencing approach (Fig. 5). Principal component analysis of all samples revealed distinct clustering of treatment groups on both days, indicating the induction of distinct phenotypes (Fig. 5a). In LEAN condition differences between day 5 and 10 were observed. These differences were predominantly observed for hallmarks of adipogenesis (Fig. 5b). This was related to an ongoing adaption of the cells to the LEAN culture condition after completion of the model manufacturing. The hierarchical clustering of samples based on gene sets for hallmarks of NASH (i.e. free fatty acid metabolism, inflammatory response and collagen synthesis), showed a distinct clustering for NASH and LEAN hLiMTs, confirming the activation of pathways associated with NASH (Fig. 5b). The expression of genes associated with lipid metabolism (e.g. FASN, LDLR, PPARα), inflammation (e.g. IL1B, CCL2, CXCL8) and fibrosis (e.g. COL1A2, TIMP1, FN1) were analyzed and found to be up- or down-regulated on both days 5 and 10, with larger fold changes and significance levels observed on day 10 (Supplementary Fig. S2c,d). These results are in accordance with the biochemical findings, and further support the progression of the disease. Differentially expressed gene analysis and GSEA were performed (Fig. 5c and Supplementary Fig. S2a,b). GSEA revealed a strong regulation of metabolic and inflammatory pathways, further confirming the induction of the NASH phenotype. There was a regulation of many metabolic pathways, including CYPs, which is in line with studies showing a dysregulation of CYPs in NASH patients^53^.

### Proof-of-concept studies: recapitulation of clinical and mechanistic findings

#### Anti-steatotic effect of Firsocostat (ACCi)

**Figure 6:**
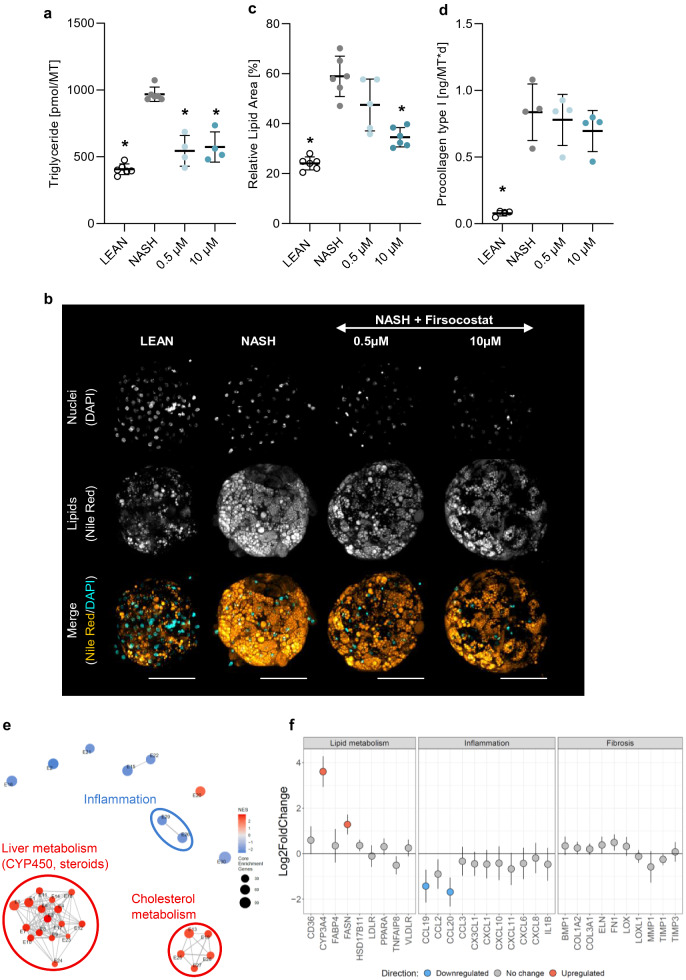
Firsocostat treatment leads to decreased lipid accumulation in NASH hLiMTs. (**a**) Firsocostat significantly decreased TG levels compared to NASH control hLiMTs. (**b**) Confocal images of Nile Red stainings demonstrated a decrease in the accumulation of the lipids in Firsocostat-treated NASH hLiMTs compared to NASH control hLiMTs. (**c**) Quantification of lipid content using HCI confirmed the anti-steatotic effect of Firsocostat and showed a reduction of the relative lipid area at 0.5 µM and 10 µM Firsocostat (*p < 0.05). (**d**) For Firsocostat a mild, concentration dependent but not significant reduction in procollagen type I secretion was observed. (**e**, **f**) Transcriptomic profiling of Firsocostat treatment (10 µM) in the 3D NASH model. (**e**) Enrichment map of gene sets derived from GSEA comparing Firsocostat-treated NASH hLiMTs to NASH hLiMTs on day 10 of treatment. The 30 most significantly regulated gene sets are displayed. Color scale represents NES. Red nodes represent gene sets upregulated upon Firsocostat treatment, whereas blue nodes represent gene sets downregulated. The size of the nodes depicts the numbers of core enrichment genes. Nodes are grouped by their similarity, with connecting lines representing common genes between nodes. Cluster of functionally related nodes were summarized and annotated. Full gene set lists are provided in Supplementary Table S3. (**f**) Expression profile of key genes associated with lipid metabolism, inflammation and fibrosis. Plots show log2 fold change between Firsocostat-treated NASH hLiMTs and non-treated NASH hLiMTs on day 10 of treatment. Error bars represent 95% confidence intervals associated with log2 fold change values. Blue indicates significant downregulation and red significant upregulation in Firsocostat-treated NASH samples (FDR < 0.05). Data points are derived from single hLiMTs (n = 5–10).

The anti-steatotic and anti-fibrotic effects of Firsocostat were investigated by treatment of hLiMTs with NASH conditioned medium in the presence and absence of compounds. TG levels in NASH hLiMTs were significantly (*p < 0.05) decreased by 0.5 and 10 μM Firsocostat (Fig. 6a) and comparable with those in LEAN hLiMTs. Confocal images of Nile Red-stained hLiMTs confirmed a concentration-dependent decrease in the accumulation of lipids by Firsocostat in NASH treated tissues (Fig. 6b). Firsocostat at 10 µM, significantly decreased (*p < 0.05) the accumulated lipids to a lower level than that in untreated NASH hLiMTs (Fig. 6c). The secretion of procollagen type I was mildly decreased in a concentration-dependent manner by Firsocostat, although this was only observed as a trend (Fig. 6d).

Firsocostat did not affect the secretion of the six measured pro-inflammatory cytokines/chemokines (data not shown); however, whole transcriptomic profiling of 10 μM Firsocostat-treated NASH hLiMTs on day 10 indicated down-regulation of pro-inflammatory pathways (Fig. 6e,f). There was also an upregulation of metabolism-related genes by Firsocostat (Fig. 6e,f). Interestingly, hierarchical clustering based on gene sets associated with hallmarks of NASH (Supplementary Fig. S3b) showed distinct clustering for adipogenesis, inflammatory responses and collagen formation.

#### Anti-inflammatory and anti-fibrotic effect of Selonsertib (ASK1i)

**Figure 7:**
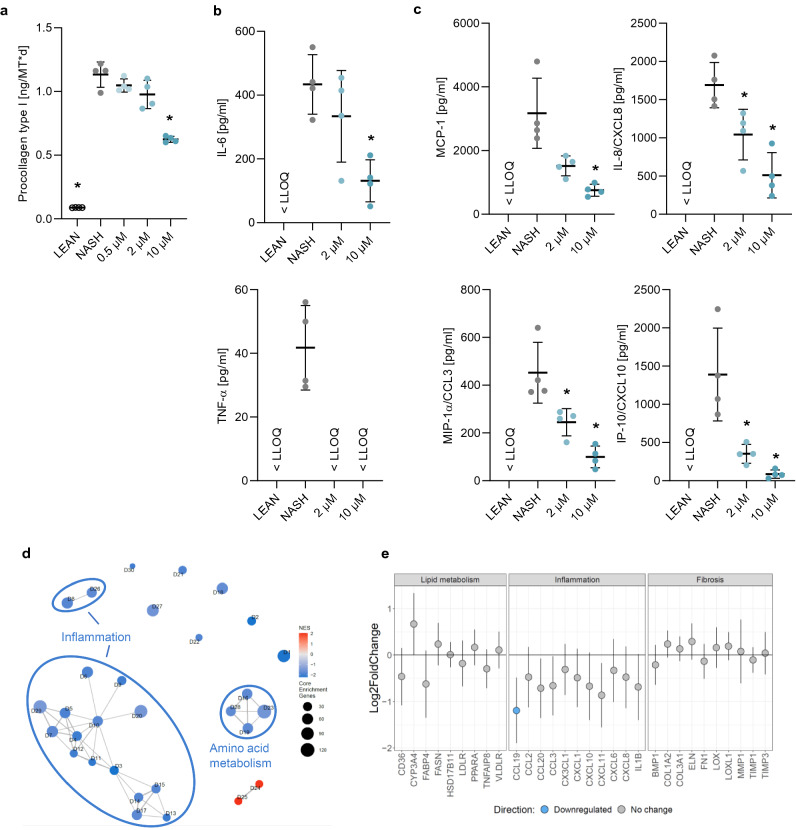
Selonsertib decreased the inflammation and fibrosis in NASH hLiMTs. (**a**) Selonsertib concentration-dependently decreased secretion of procollagen type I in the 3D NASH model. This reduction was significant at 10 µM (*p < 0.05). (**b**, **c**) Selonsertib exhibited anti-inflammatory activity, evident as a concentration-dependent decrease in the production and secretion of (**b**) pro-inflammatory cytokines, TNF-α and IL-6 and (**c**) chemokines, MCP-1, MIP-1α (CCL3), IP-10 (CXCL10) and IL-8 (CXCL8). (**d**, **e**) Transcriptomic profiling of Selonsertib treatment (2 µM) in 3D NASH model. (**d**) Enrichment map of gene sets derived from GSEA, comparing Selonsertib-treated NASH samples to non-treated NASH hLiMTs on day 10 of treatment. The 30 most significantly regulated gene sets are displayed. Color scale represents NES. Red nodes represent gene sets upregulated upon Selonsertib treatment, whereas blue nodes represent gene sets downregulated. The size of the nodes depicts the numbers of core enrichment genes. Nodes are grouped by their similarity, with connecting lines representing common genes between nodes. Clusters of functionally related nodes were summarized and annotated. Full gene set lists are provided in Supplementary Table S4. (**e**) Expression profile of key genes associated with lipid metabolism, inflammation and fibrosis. Plots show log2 fold change between Selonsertib-treated NASH samples compared to non-treated NASH hLiMTs on day 10 of treatment. Error bars represent 95% confidence intervals associated with log2 fold change values. Blue indicates significant downregulation and red significant upregulation in Selonsertib-treated NASH samples (FDR < 0.05). Data points are derived from single hLiMTs (n = 5–10).

The anti-inflammatory and anti-fibrotic effects of Selonsertib were investigated by incubating hLiMTs in NASH induction medium in presence and absence of the drug. As expected, there was no effect of Selonsertib on tissue TG levels (data not shown). However, the anti-fibrotic effect of Selonsertib was evident as a concentration-dependent and significant decrease the synthesis and secretion of procollagen type I (Fig. 7a). Anti-inflammatory activity of Selonsertib was reflected as a concentration-dependent decrease in the secretion of pro-inflammatory cytokines: TNF-α and IL-6 (Fig. 7b) and chemokines: MCP-1, MIP-1α, IL-8, IP-10 (Fig. 7c).

Whole transcriptomic profiling revealed that Selonsertib treated (2 μM) hLiMTs (Fig. 7d,e) resulted in a strong down-regulation of pro-inflammatory pathways and decreased expression of key pro-inflammatory markers on day 10, in alignment with the biochemical analyses. According to hierarchical clustering based on gene sets associated with hallmarks of NASH, there was a distinct clustering for adipogenesis, inflammatory responses and collagen formation (Supplementary Fig. S3c).

### Prevention of fibrosis in NASH hLiMTs by ALK5i

**Figure 8:**
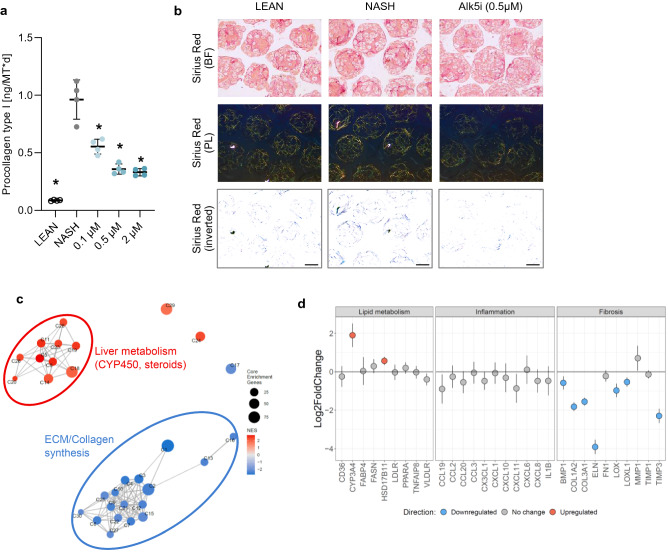
Effect of ALK5i on fibrosis progression in NASH hLiMTs. (**a**) Procollagen type I secretion was quantified in the supernatants on days 7 and 10. ALK5i decreased secretion concentration-dependently, (0.1 µM, 0.5 µM, 2 µM, *p < 0.05). (**b**) Polarized light microscopy of Sirius Red-stained tissue sections. There was a reduction of collagen fibrillation in ALK5i-treated NASH hLiMTs compared to non-treated NASH hLiMTs. Scale bars indicate 100 μm. (**c**, **d**) Transcriptomic profiling of ALK5i treatment (0.5 µM) in the 3D NASH model. (**c**) Enrichment map of gene sets derived from GSEA comparing ALK5i-treated NASH hLiMTs to non-treated NASH hLiMTs on day 10 of treatment. The 30 most significantly regulated gene sets are displayed. Color scale represents NES. Red nodes represent gene sets upregulated upon ALK5i treatment, whereas blue nodes represent gene sets downregulated. The size of the nodes depicts the numbers of core enrichment genes. Nodes are grouped by their similarity, with connecting lines representing common genes between nodes. Clusters of functionally related nodes were summarized and annotated. Full gene set lists are provided in Supplementary Table S5. (**d**) Expression profile of key genes associated with lipid metabolism, inflammation and fibrosis. Plots show log2 fold change between ALK5i-treated NASH hLiMTs compared to non-treated NASH hLiMTs on day 10 of treatment. Error bars represent 95% confidence intervals associated with log2 fold change values. Blue indicates significant downregulation and red significant upregulation in ALK5i-treated NASH samples (FDR < 0.05). Data points are derived from single microtissues (n = 10).

The anti-fibrotic effects of ALK5i were investigated by incubating hLiMTs in NASH induction medium in presence and absence of the drug. There was a concentration-dependent decrease in the synthesis and secretion of procollagen type I by ALK5i (Fig. 8a). Furthermore, decreased deposition of collagen fibrils was visualized under bright field and polarized light on histological sections stained with Sirius Red (Fig. 8b).

As expected, Alk5i had no effects on TG accumulation, however an increase of cytokine/chemokine release was seen (data not shown), which can be explained by a reduction of TGF-β mediated anti-inflammatory effects^54^.

The results of the biochemical analyses were further confirmed by whole transcriptomic profiling of ALK5i (0.5 μM)-treated NASH hLiMTs on day 10. There was a strong down-regulation of pathways associated with collagen synthesis and ECM production (Fig. 8c), as well as a significant decrease in key fibrotic genes, such as COL1A2, COL3A1, ELN and TIMP3 (Fig. 8d). Hierarchical clustering based on gene sets associated with hallmarks of NASH revealed a distinct clustering for adipogenesis, inflammatory responses and collagen formation (Supplementary Fig. S3d).

## Discussion

There is an unmet need for development of human-relevant NASH models that can recapitulate key hallmarks of the disease, facilitate mechanistic studies, and allow rapid assessment of various modalities or combinatorial treatments. Here we demonstrated a complex 3D liver model suitable for NASH disease modeling. We have characterized a novel primary 3D human liver model that incorporates relevant liver cell types for investigating NASH. The maintained presence of the liver cells critical to NASH: PHHs, LECs, KCs, and HSCs, were confirmed throughout the treatment period by positive staining of cell-specific markers. To generate a phenotype that mimics NASH, the non-parenchymal cells were required to ensure inflammatory state and HSCs to facilitates liver fibrosis. The different cell types were individually added to the model, which facilitates the potential to explore how the ratio of the different cell types affects the disease phenotype. The PHHs represent a pool of ten donors, whereas the KC/LECs and HSCs derived from single donors. The protocol allowed for LEAN as well as NASH hLiMTs; the latter by supplementing the medium with elevated sugars, FFA, and a pulse of LPS. This complex and clinically-relevant stimuli resulted in hLiMTs displaying all three major clinical hallmarks of NASH. Both micro- as well as macrovesicular steatosis were evident through Nile Red staining of lipids, accumulation of TGs, and displacement of nuclei. Induction of inflammatory pathways was detected by a substantial increase of cytokine and chemokine secretion into the media. Recent review articles have highlighted a key subset of the cytokines/chemokines observed in the clinical disease^51^ and most of them were increased in our model. A significant amount of fibrosis was achieved and represented by both secretion of procollagen type I, as well as increased collagen deposition and fibrillation. Not only were biochemical endpoints (TG, cytokines/chemokines, and PCI) achievable in these LiMTs, but they also exhibit an impressive dynamic range, which allows for ranking of compound effects on all three hallmarks of NASH. Whole transcriptomic profiling also clearly distinguished between LEAN and NASH treated hLiMTs. Significant up-regulation of genes related to inflammation and fibrosis, as well as down-regulation of genes related to lipid metabolism, reaffirmed the biochemical processes and progression known to occur in NASH. The gene expression of a range of cytokines/chemokines such as interleukin-1β (IL-1β), chemokine (C–X–C motif) ligand 6 (CXCL6), C–C motif chemokine ligand 20 (CCL20), was increased by the loading of cells with FFAs and LPS (Supplementary Fig. S2d). Furthermore, the fibrosis biomarkers such as tissue inhibitor of metalloproteinases 1 (TIMP-1), collagens, fibronectin and elastin (Supplementary Fig. S2d) were upregulated on gene expression level^2,55^.

In order to recreate NASH disease progression within a short exposure time, optimal medium conditions have been critical. NAFLD is closely linked with obesity and diabetes^56^, and is therefore the basis of the stimuli used in the NASH hLiMT model described here. NASH is thought to be initiated by fatty acid accumulation and endotoxin derived inflammation, resulting in a cascade of inter-linked processes that cause oxidative stress, fatty acid oxidation, mitochondrial dysfunction and, ultimately, a pro-inflammatory state that leads to fibrosis^12,56^. We have modeled these processes in the short NASH induction phase by incubating the hLiMTs under diabetic conditions, lipotoxic and inflammatory stress, rather than with the inflammatory cytokines themselves. Others have investigated in vitro NASH models using strong stimuli like TGF-β and showed that simply adding this potent pro-fibrotic mediator resulted in poorer fibrotic phenotype compared to that induced by supplementing the medium with FFA^13^. Overstimulation of in vitro models might affect cell viability, as well as reduce the response of the model to compound effects. Although LPS is also considered a strong stimulus, it reflects the clinical situation as it is measured in the blood or NASH patients, likely a result of gut microbiota alterations and bacterial translocation^57^. Others have used a very high concentration of palmitic acid (0.5 mM) to mimic NASH in multicellular InSphero 3D InSight™ 3D liver microtissues^23^; however, such high concentrations are not observed clinically. In their model, although high palmitic acid concentrations induced high toxicity and tissue damage, as well as several markers of the inflammatory (e.g., IL-8) and pro-fibrotic process (e.g., TIMP-1, PDGFRβ, collagen I and III), palmitic acid did not induce lipid accumulation or a steatosis-like phenotype.

In addition to the stimuli used in an in vitro NASH model, the cell type and culture format are also crucially important. The most commonly used 2D human cellular models (e.g. immortialized cells, primary hepatocyte monocultures) fall short due to diminished longevity and the absence of critical hepatocyte-NPC interactions required for NAFLD progression^58^. Some researchers have combined immortalized cells e.g., HepG2 and LX2, to represent hepatocytes and stellate cells, respectively^59^. However, the use of immortalized cells does not allow for investigation of donor variations and introduces uncertainties with respect to their genotype^14^. HepG2 cells have the disadvantage of lacking sufficient metabolic capacity, while HepaRG cells represent an alternative hepatocyte type that exhibits a more comparable metabolizing capacity to PHHs^60^. Although they are also immortalized cells from a single donor, HepaRG cells have been used to investigate mechanisms involved in NASH, either in the absence of NPCs in 2D culture^19^ or in combination with other liver cell types^13,61^. These models tend to only represent one part of the NASH pathogenesis e.g., steatosis^19^ or fibrosis only^61^. As an alternative to cell lines, some researchers have incorporated extra-hepatic primary cells, such as HUVECs^13^ to the other liver cell types in the 3D tissue model; however, these might not fully mimic liver specific processes and cell–cell interactions, which is crucial to understand initiation and progression of a complex disease like NASH. It has been shown that spheroidal 3D tissue model composed of mixture of transformed cell lines: HepaRG and HUVEC as well as primary KC and HSC, developed lipid accumulation, inflammation and fibrosis upon treatment with FFA^13^. Recently has been reported that human hepatic 3D spheroid system consisting of PHH cultured with free fatty acids and insulin mimic the steatotic conditions in a reversible manner^62^. Similar spheroidal model of PHH in co-culture with crude fractions of NPC from several matched or non-matched donors, has been shown to display a fibrotic phenotype either spontaneously, primarily observed in patatin like phospholipase domain containing 3 (PNPLA3) mutant donors, or after challenge with FFA, as determined by COL1A1 and αSMA expression^63^. In this model, however, no inflammatory process as a hallmark of NASH was observed.

The NASH model described in the current study involves the formation of hLiMTs composed of multi-donor PHHs in co-culture with primary HSCs and KCs and LECs. All these liver cell types are crucial for disease development, and enhance the in vivo translational relevance of the NASH model. Through the 3D incorporation/communication between human relevant primary cells derived from the liver, and donor variability minimized by using ten multi-donor PHHs lot, we are able to overcome the limitations seen in immortalized cell lines and individual donor lots of primary cells. Compound efficacy testing in a multi-donor PHH lot represents a larger average population, preventing the potential biased conclusion from a single human donor. To overcome the problems associated with allogeneic reaction of mixing multi-donor lot of PHH with multi-donor lots of KCs, LEC and HSC only single donors of NPCs were used. For the initial establishment the 3D NASH hLiMTs, several KCs and LECs lots were tested and the ones with no allogeneic reaction, but with good inflammatory and fibrosis response were chosen for further model development. In contrast to flow-based microphysiological systems (MPS) with limited throughput capacity^64,65^, the 96-well format of the hLiMT model is scalable and enables the testing of numerous compounds, as well as combinations of compounds. Furthermore, current models with MPS and hemodynamics conditions have been shown to contain only PHHs, KCs (macrophages) and HSCs, but lack the important LECs^22,64,65^. LEC have been shown to pay pivotal role in provoking fibrosis through stromal cell-derived factor I receptors CXCR7/CXCR4 and fibroblast growth factor receptor 1 (FGFR1)^2^. Similarly to our NASH model, the other also reported an in vitro spheroidal NASH model, which incorporates PHHs, KC, LEC and HSC. A NASH-like phenotype has been induced by addition of medium containing FFA and TNF-α for treatment period of 10 days. In contrast to studies presented here, the authors reported only low level of steatosis, inflammation and early fibrosis. No increased extracellular collagen fibers deposition and formation was demonstrated on histological level^55^.

Perhaps the most important aspect of research model systems is the ability to recapitulate compound effects observed in the clinic. A prime example of predictivity is the use of the 3D liver model for toxicity assessment, which has been validated by pharma with a set of more than 100 compounds^66^. Once more clinically proven drugs are available for testing, a similar validation study on the NASH model can further underline its value in recapitulating the disease. Nevertheless, advanced model systems can help to further understand MoAs of compounds and deconvolute processes like cellular crosstalk and cell matrix interaction. As a first step towards such predictive models, we performed proof-of-concept experiments to analyze the effect of clinical drug candidates in our model system and compared them to results from the clinic. Firsocostat has been shown not only to decrease the de novo lipogenesis, but directly to suppress TGF-β-induced activation of HSC^6^. The anti-steatotic effects of Firsocostat were evident as a reduction of TG in NASH hLiMTs. This effect was confirmed by image-based quantification of lipids, correlating with the effect observed by magnetic resonance imaging-estimated proton density fat fraction (MRIPDFF) diagnostics in patients taking part in a clinical study^49^. Interestingly, Firsocostat did not decrease lipids to LEAN control level. The rationale for this observation could be that in our model, cells are constantly kept at a nutrient rich “fed” state, thus keeping beta-oxidation at an already low level. Firsocostat has been shown to have anti-fibrotic effects, which was nicely recapitulated in our model as a dose-dependent decrease in procollagen type I production. Interestingly, while Firsocostat did not affect the secretion of the six measured pro-inflammatory cytokines/chemokines, there was a down-regulation of other markers such as cytokines CCL19, CCL20 and CCL21 at the gene expression level^25^. In this case, the gene expression profile provided additional information on the MoA of Firsocostat, which is advancing in clinical combination studies with a Farnesoid X receptor (FXR) agonist and glucagon-like peptide-1 receptor (GLP-1R) agonist highlighting the importance of combined and multiparametric endpoints^67^. The maximum serum concentration (C_max_) in patients treated with Firsocostat has been shown to be 5.4 ng/ml (9.5 nM) at 2–3 h post-dosing with the compound^68^. This concentration is lower that the effective tested concentrations of Firsocostat (0.5 µM and 10 µM) in NASH hLiMT. Other studies also demonstrated an effect of 0.5 µM and 1 µM Firsocostat on inhibition of fibrosis in LX-2 cells and primary HSC^29^. This difference in the compound concentrations found in the patient plasma and the one used in the 3D NASH efficacy testing could be based on different treatment schemes in clinic versus in vitro cultures, or might be due to lower, unbound, Firsocostat concentration in 3D NASH model.

The anti-inflammatory and anti-fibrotic effects of the ASK1 inhibitor Selonsertib were demonstrated in the NASH hLiMTs. In line with early clinical findings^69^, anti-inflammatory effects were evident as a decrease in the secretion of pro-inflammatory cytokines and chemokines, as well as at the gene expression level, with a strong down-regulation of pro-inflammatory pathways. The anti-fibrotic mechanism the most relevant clinical endpoint for improving patients’ health condition was determined in the hLiMTs as a decrease in procollagen type I secretion^69^. Interestingly, our results are in accordance with the results of a phase II clinical trial, in which Selonsertib was administered for 24-weeks and resulted in improvement in fibrosis among NASH patients with moderate to severe fibrosis F2–F3. However, in the phase III clinical trial, in which Selonsertib was administered for 48 weeks, there was not a significant anti-fibrotic effect observed in NASH patients with bridging fibrosis F3 or compensated cirrhosis F4^70^. In first-in-human clinical studies, the EC_50_ in human whole blood was determined to be 56 ng/mL (0.13 μM)^71^, which is lower than the concentrations tested in the NASH hLiMTs model (2 and 10 μM). Thus, the concentrations tested here are in the pharmacologically relevant clinical range. This suggests that our NASH model most likely reflect the F2–F3 clinical stages of fibrosis, where we see a prominent anti-fibrotic effect of Selonsertib. The clinical C_max_ of Selonsertib has been shown to be 40.5 or 3640 ng/ml (0.09–8.17 µM) dependent on the applied compound dose 1 or 100 mg, respectively^71^. These clinical C_max_ concentrations are very closed to the effective tested concentrations of Selonsertib (2 µM and 10 µM) in NASH model. The clinical Cmax of Selonsertib has been shown to be between 40.5 and 3640 ng/mL (0.09–8.17 µM) dependent on the applied compound dose 1–100 mg^71^. These clinical C_max_ concentrations are very closed to the effective tested concentrations of Selonsertib (2 µM and 10 µM) in NASH hLiMT. Other studies also previously demonstrated an effect of 1 µM Selonsertib on inhibition of fibrosis and inflammation in palmitate-induced 3D NASH microtissue models^23^. ALK5i is not in clinical development but was used as a model compound to manipulate TGF-β signaling and the downstream impact on HSCs and fibrosis. There was a clear effect of ALK5i observed on the fibrotic phenotype of the NASH model, including a reduction of procollagen I release and deposition of collagen, as well as a down-regulation of fibrotic pathways and genes. The findings from ALK5i treatment indicate the presence of endogenous TGF-β signaling in the model, most likely induced by the lipotoxic and inflammatory stress. TGF-β has a stronger affinity to the matrix and it is not present in a free form. Therefore, determination of the level of secreted form of TGF-β in the supernatants of matrix rich NASH samples is difficult^72^. These findings also demonstrated that the model can help to elucidate the MoA of a candidate drug and its potency effects on NASH hLiMTs. Since the blocking and/or resolution of fibrosis is perhaps the most critical goal of NASH therapeutics, these data support the use of the 3D NASH model as a suitable pre-clinical drug discovery tool to assess anti-fibrotic effects of compounds.

In conclusion, we have developed a 3D NASH model, which incorporates all relevant primary human liver cell types, shown previously to play a role in the mechanisms of disease induction mediated by complex cellular crosstalk^14^. NASH hLiMTs can recapitulate the key hallmarks of NASH and in proof-of-concept studies, we have recapitulated the effects of drug candidates on lipid accumulation, inflammation and fibrosis in this disease model.

Despite these very promising results, further development of the NASH model is needed, since some aspects of the disease and targets for drug therapy are not present, e.g., peripheral immune cell infiltration. Since this is an important step in disease progression, the implementation of peripheral blood mononuclear cells may provide an additional complexity that helps identify new therapies. Moreover it will allow to assess the later stages of NASH progression and eventually model the reversion of the disease where peripheral blood macrophages play a relevant role for tissue remodeling^73^.

A second aspect is to be able to recapitulate more advanced stages of fibrosis and employ quantification methods that can be aligned with clinical diagnostic markers. To this end, we are currently working on testing N-terminal procollagen type III peptide (PIIINP) as an additional in vitro marker that is also used in clinics^74^, as well as the enhanced liver fibrosis (ELF) test^75^. Still the gold standard for assessment of NASH in the clinic is based on histology using liver biopsy slices. Therefore, the development of phenotypic quantification of fibrosis also using histology tissue slices in the NASH model is of high priority. For assessment of the translational relevance of the NASH hLiMTs model to the in vivo situation comparison experiments will be performed using liver tissue slices of clinical and rodent NASH.

## Conclusion and outlook

We demonstrated that this novel model system mimics key features of NASH pathology such as lipid loading, inflammation, and fibrosis. These studies describe the proof-of-concept validation of anti-steatotic, anti-inflammatory and anti-fibrotic compounds, and showed that our model can recapitulate compound effects observed in animal models and clinical studies. While there are additional investigations and developments to be completed, the studies shown here demonstrate an advanced human model system for mimicking NASH. We thus propose this powerful human NASH model for drug efficacy screening and investigation of the MoA of novel drug candidates.

## Supplementary Information

Supplementary Information.
