# Supplementary material for: A 3D primary human cell-based in vitro model of non-alcoholic steatohepatitis for efficacy testing of clinical drug candidates
Source: Sci Rep. 2021 Nov 23;11:22765. doi: 10.1038/s41598-021-01951-7 (PMC8611054; doi:10.1038/s41598-021-01951-7)
Supplement: Supplementary file 1 — Supplementary Information. [file 41598_2021_1951_MOESM1_ESM.docx]

**Supplementary Figures**

**A 3D primary cell based in vitro model of non-alcoholic steatohepatitis for efficacy testing of clinical drug candidates**

Simon Ströbel*, Radina Kostadinova*, Katia Fiaschetti-Egli, Jana Rupp, Manuela Bieri, Agnieszka Pajak, Donna Busler, Thomas Hofstetter, Katarzyna Sanchez, Sue Grepper, Eva Thoma

**Affiliation:** InSphero AG, Wagistrasse 27A, Schlieren, CH

*These authors contributed equally to this work

**Corresponding author:** Simon Ströbel simon.stroebel@insphero.com

**Supplementary Figure 1**

**Supplementary Figure 1.** Model stability over time. LDH release measured on days 3, 5, 7 and 10 of culture. Chlorpromazine (CHL) was used as a positive cell toxicity control.

**
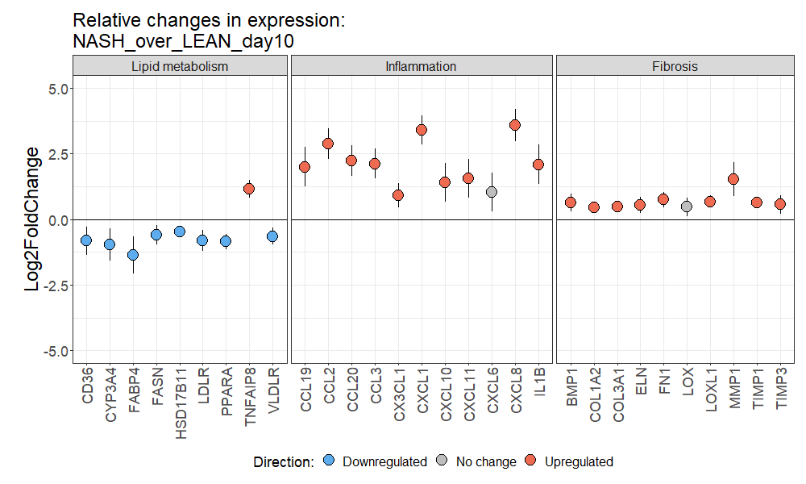

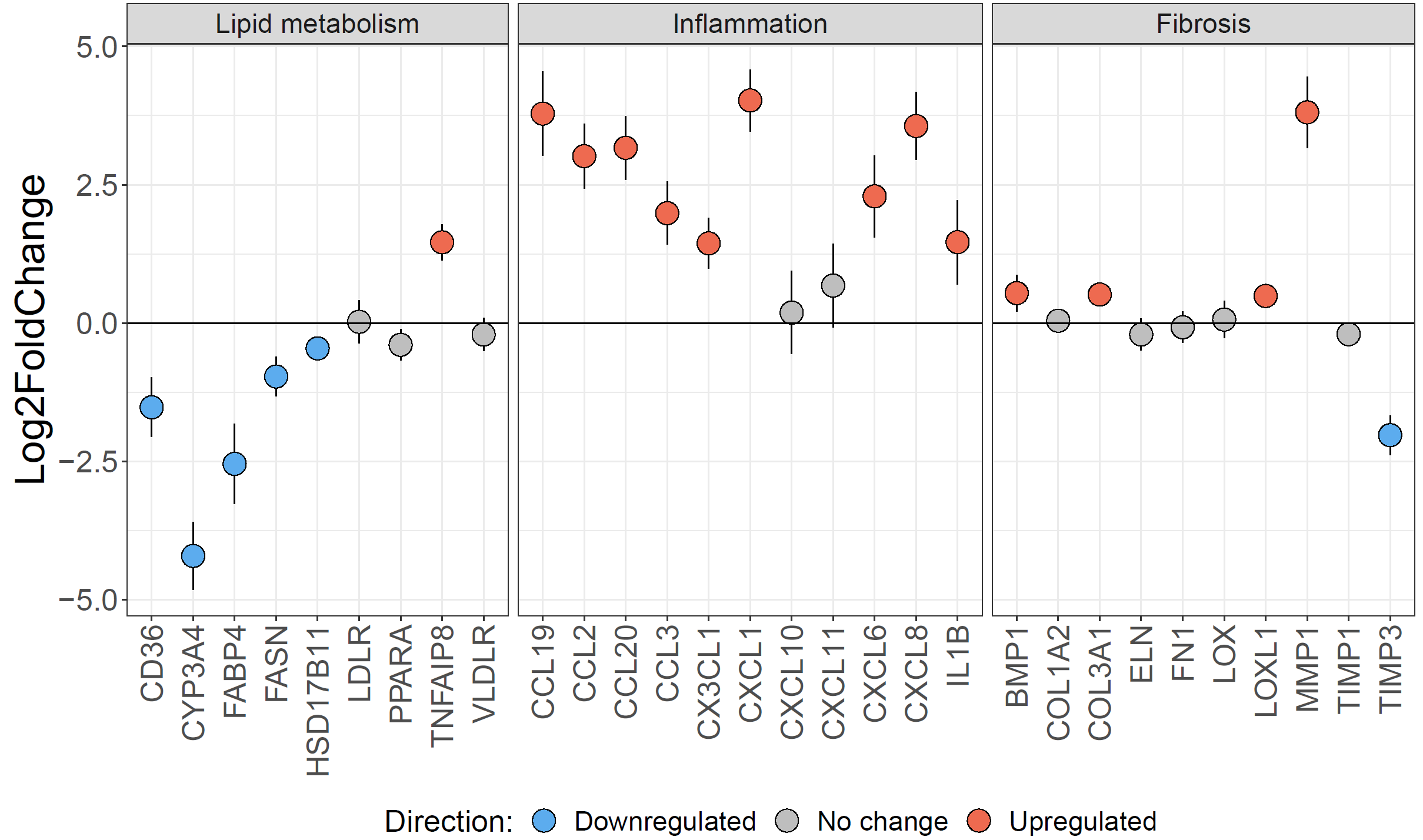

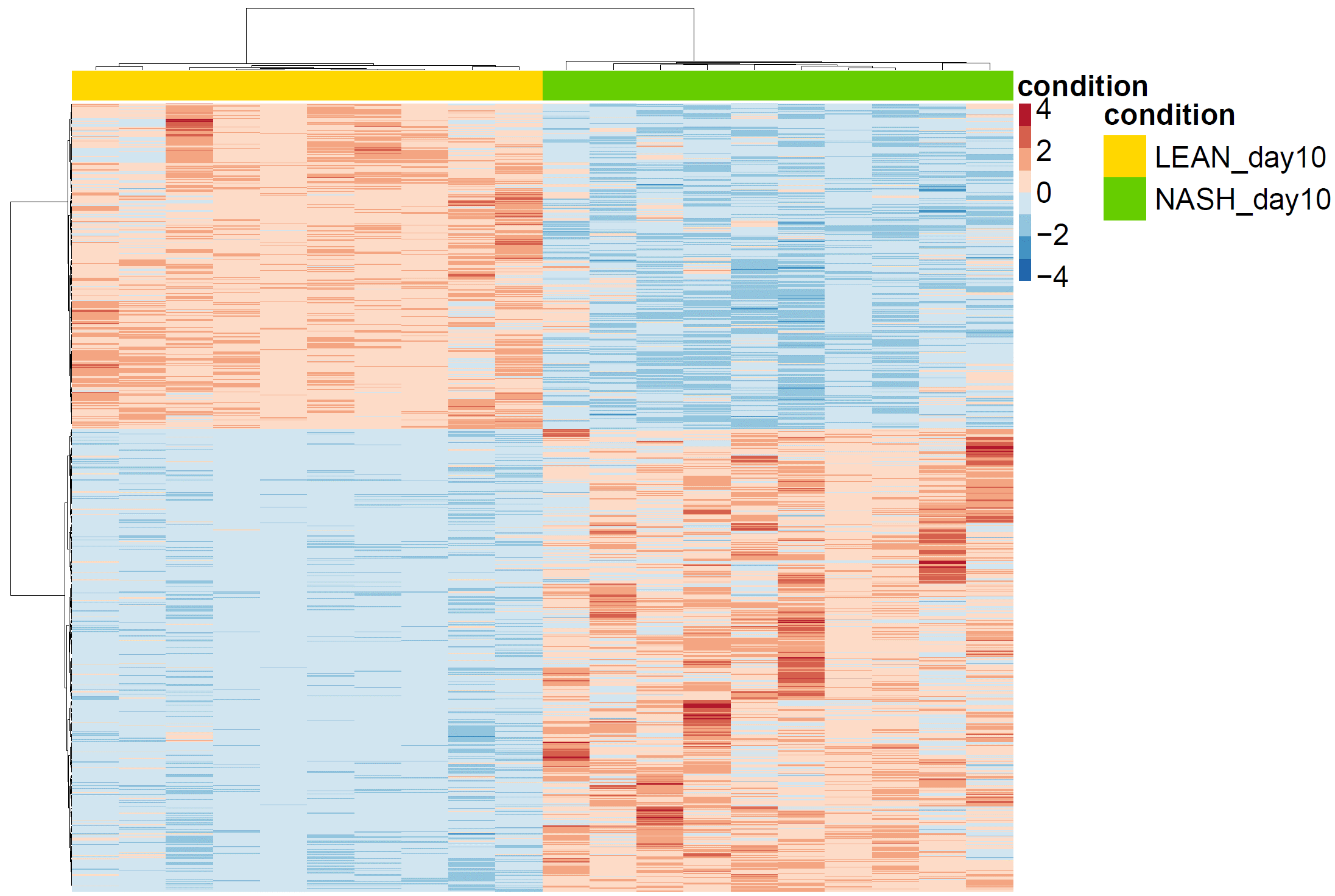

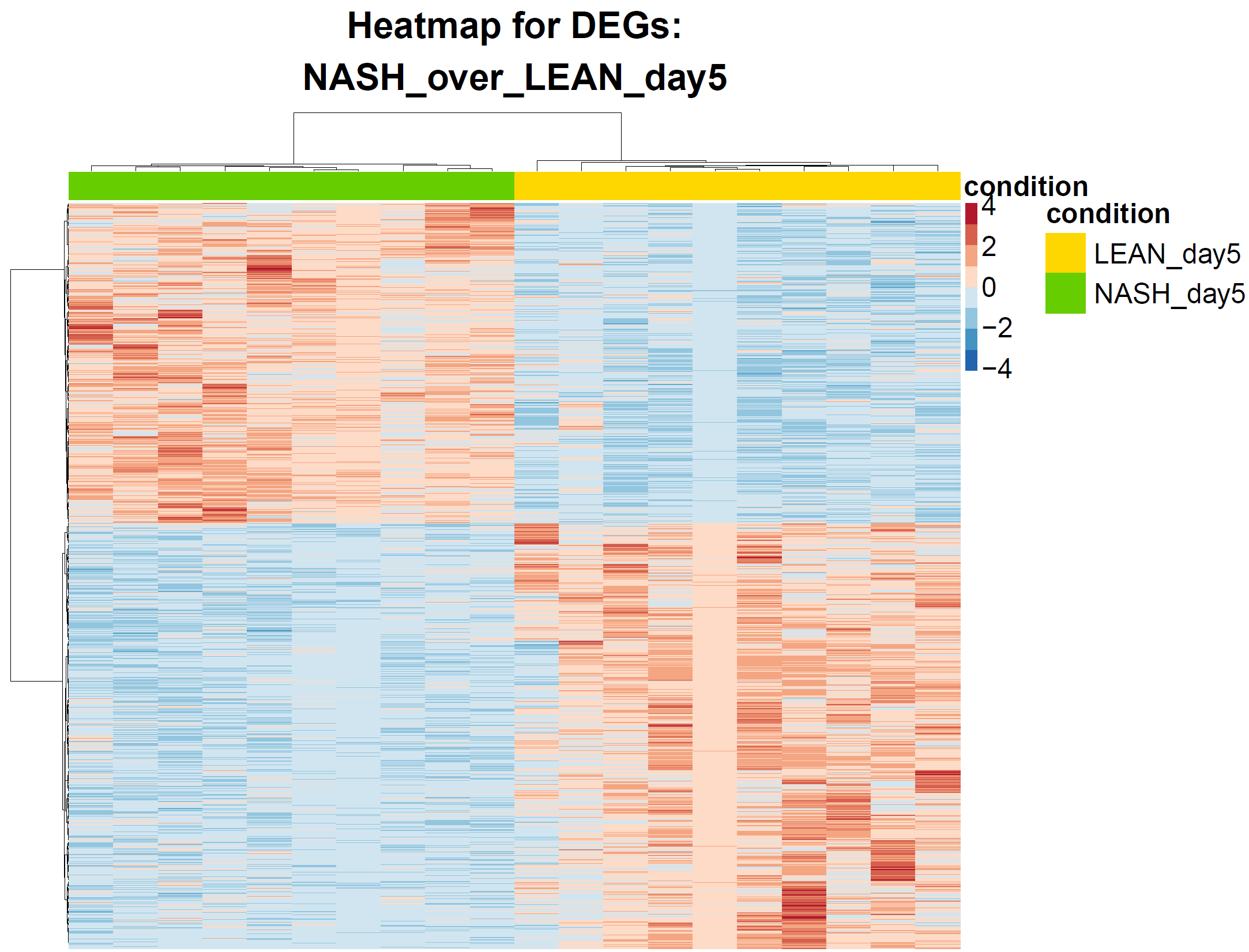
**

**Supplementary Figure 2**

**a**

**b**

**c**

**d**

**Day 5**

**Day 5**

**Day 10**

**Day 10**

**Supplementary Figure 2. Transcriptomic profiling of 3D NASH hLiMTs. (a, b)** Heatmaps for differentially expressed genes (log2 >1, FDR <0.05) between NASH and LEAN hLiMTs on day 5 **(a)** and day 10 **(b)** of treatment. Columns represent samples, and rows are genes. Row-wise Z-score transformation was performed on log2 expression values for each gene, with blue denoting a lower and red a higher expression level according to the average expression level. (**c, d**) Expression profile of key genes associated with lipid metabolism, inflammation and fibrosis. Plots show log2 fold change between NASH and LEAN hLiMTs on day 5 **(c)** and day 10 **(d)** of treatment. Error bars represent 95% confidence intervals associated with log2 fold change values. Blue indicates significant downregulation and red significant upregulation in NASH hLiMTs (FDR < 0.05). Data points are derived from single hLiMTs (n = 10), results from one of two independent experiments are shown.

**Supplementary Figure 3**

**Alk5i**

**Selonsertib**

**Firsocostat**

**a**

**b**

**c**

**d**


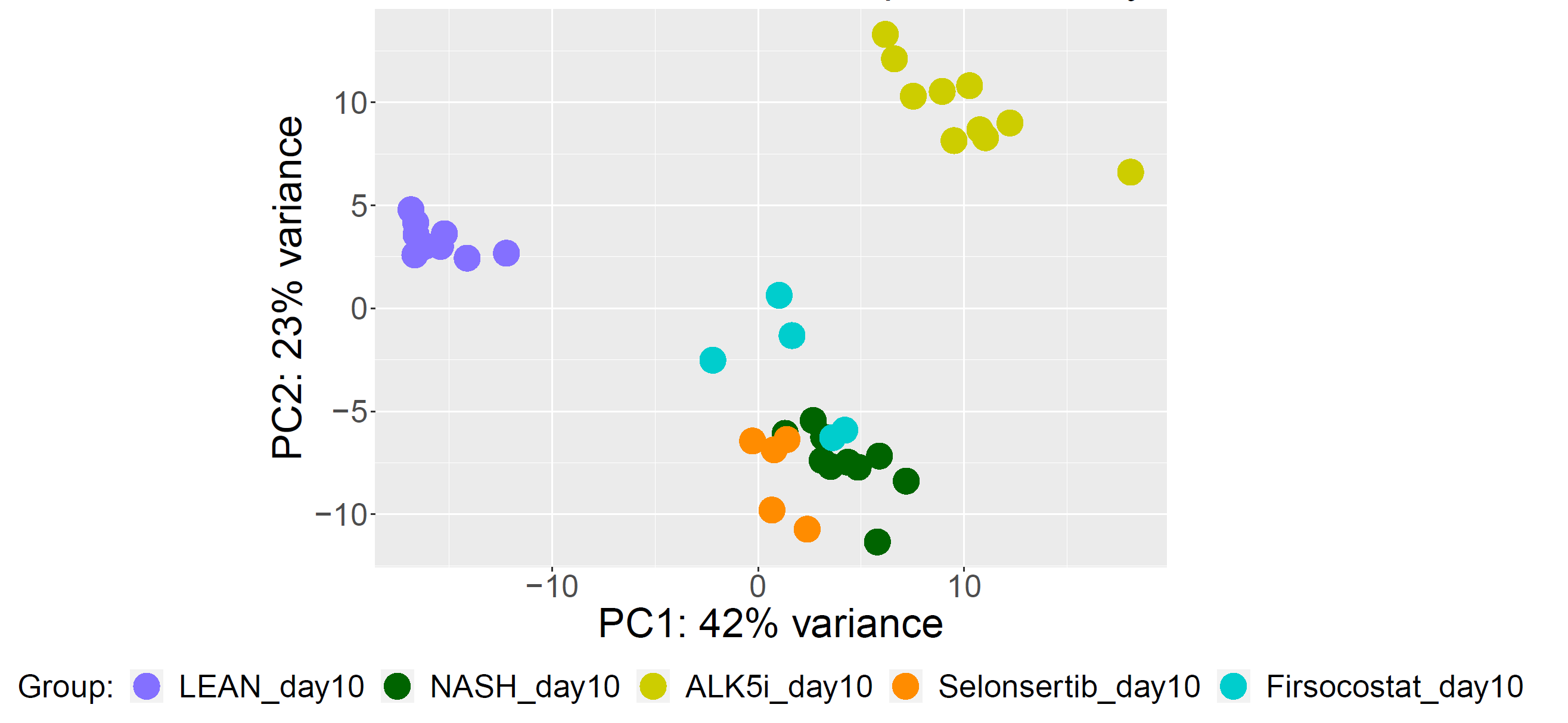

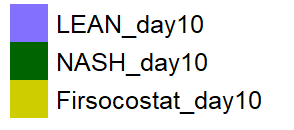

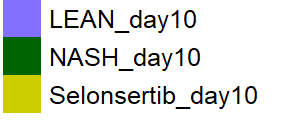

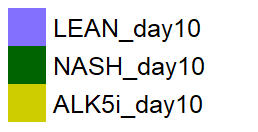


**Adipogenesis**

**Inflammatory response**

**Collagen formation**


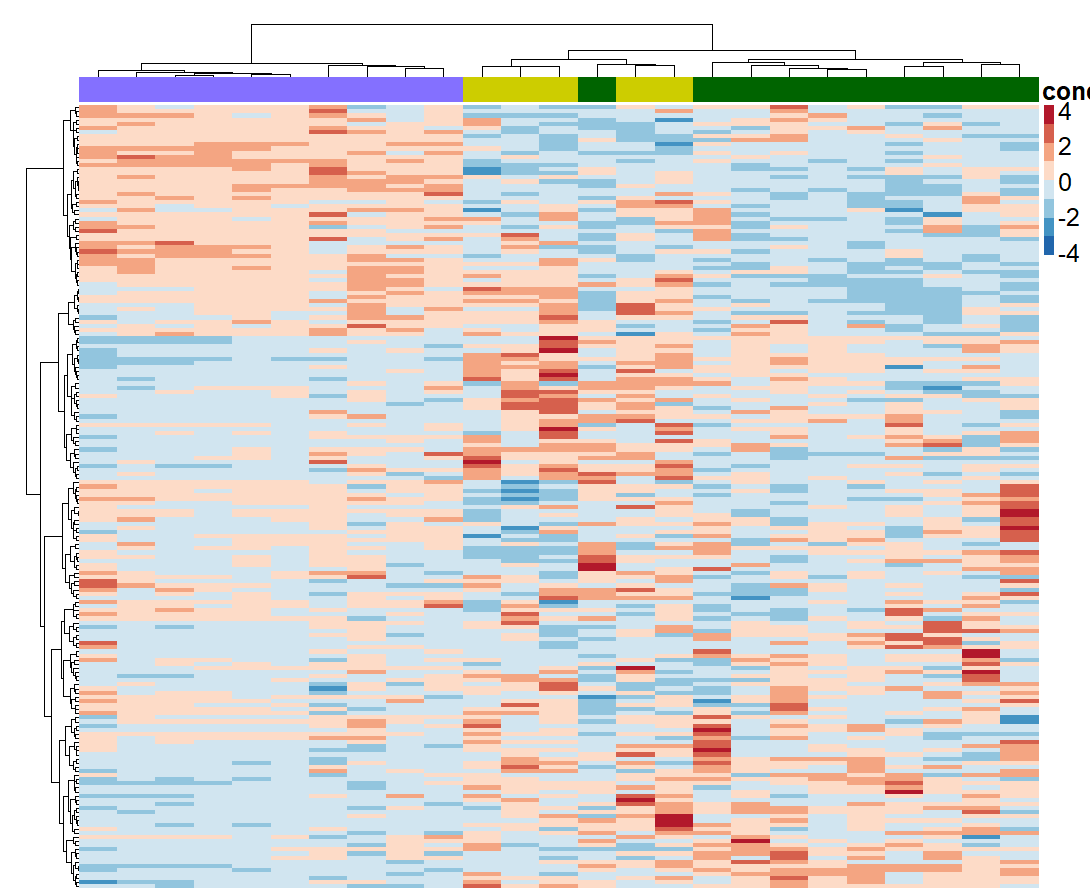

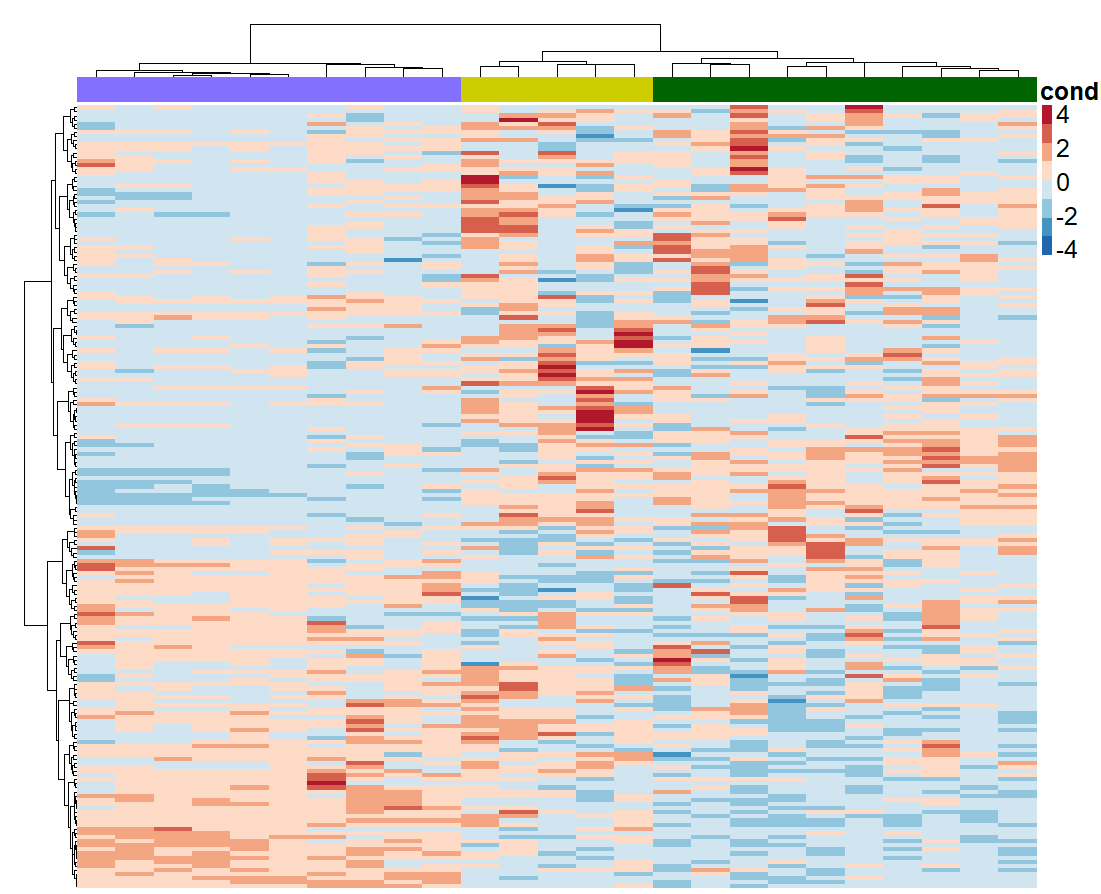

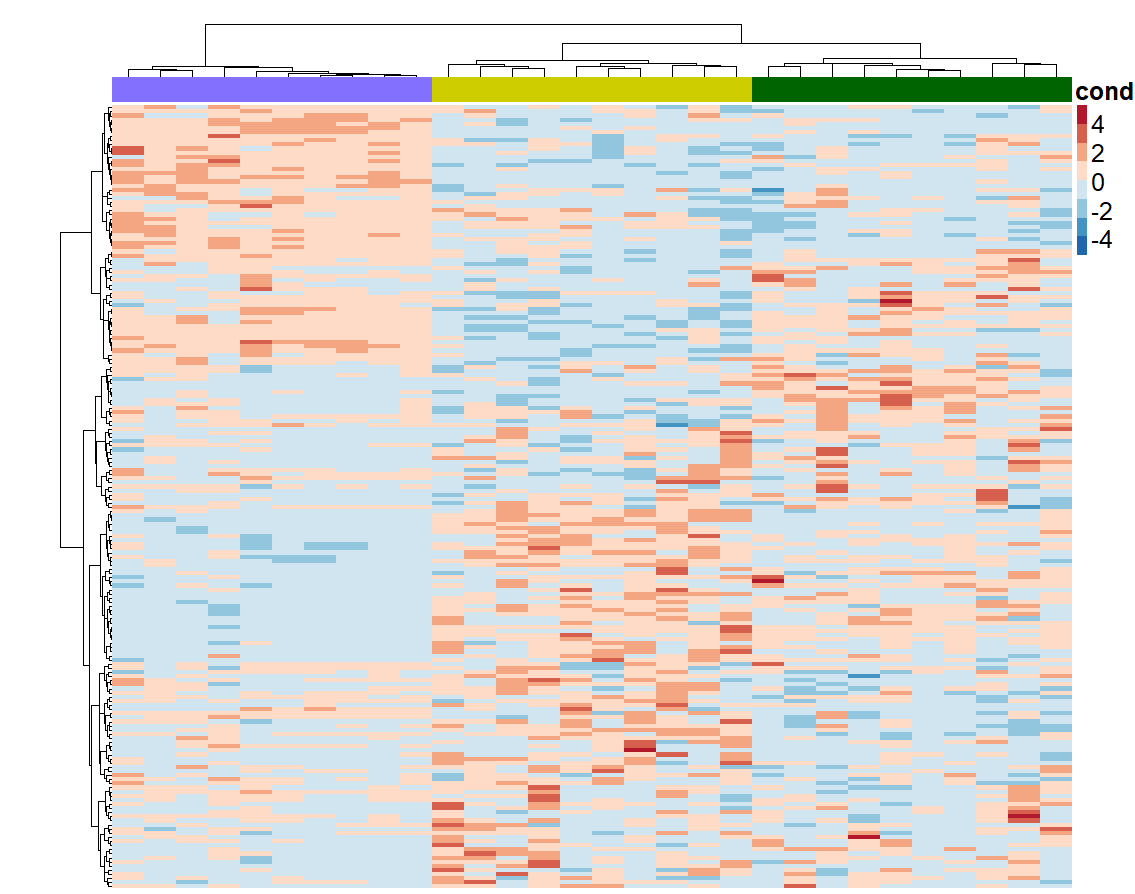

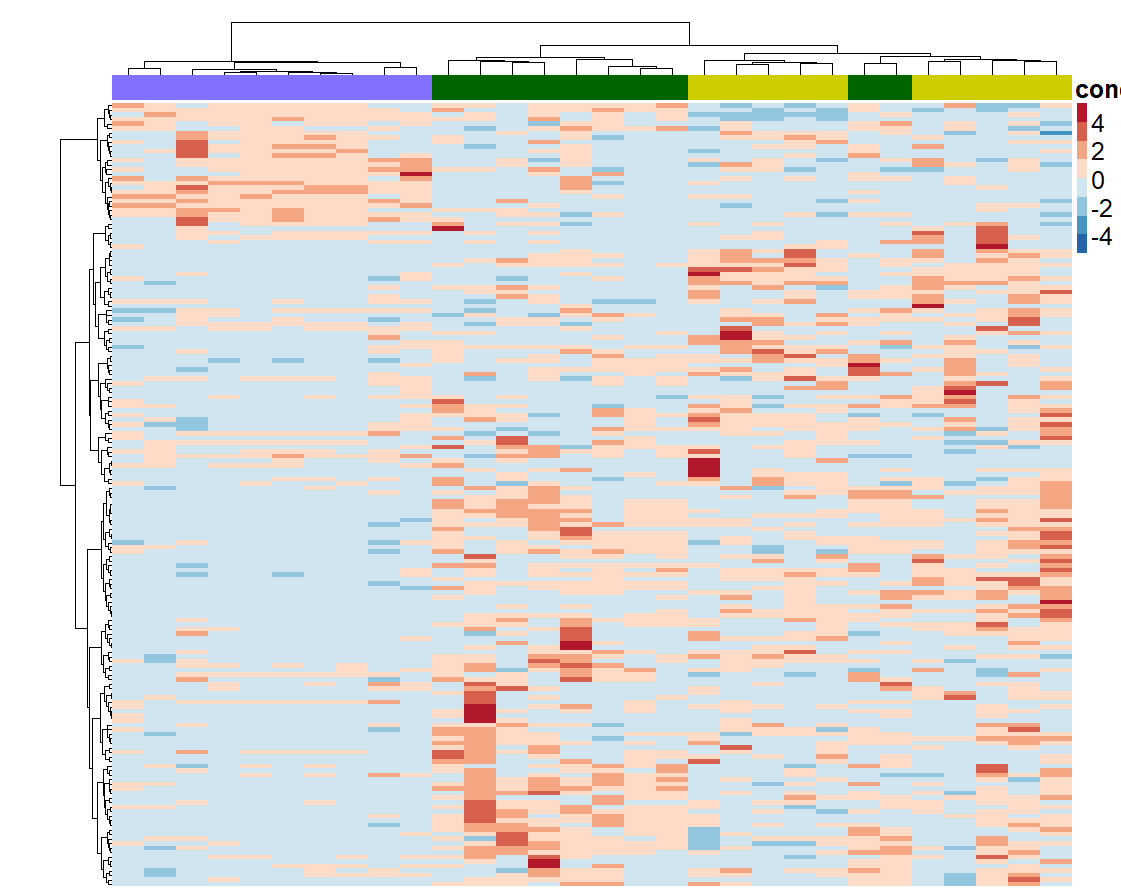

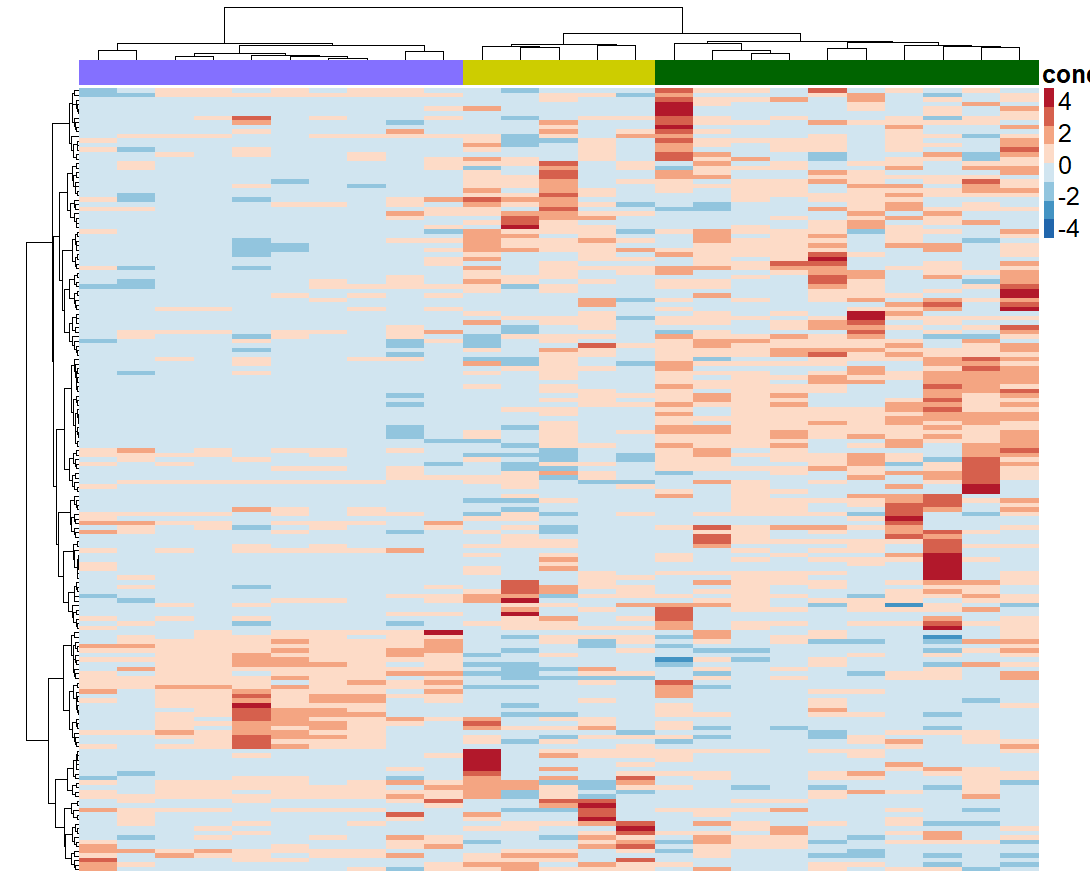

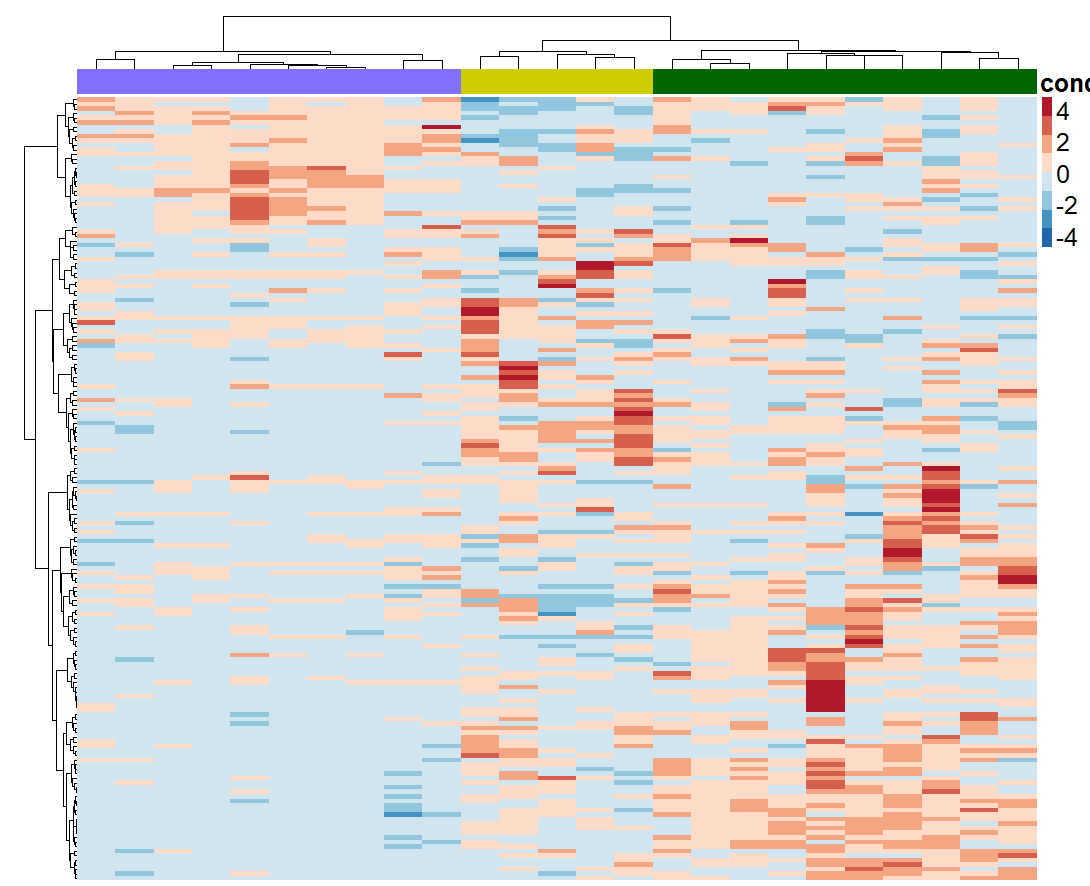

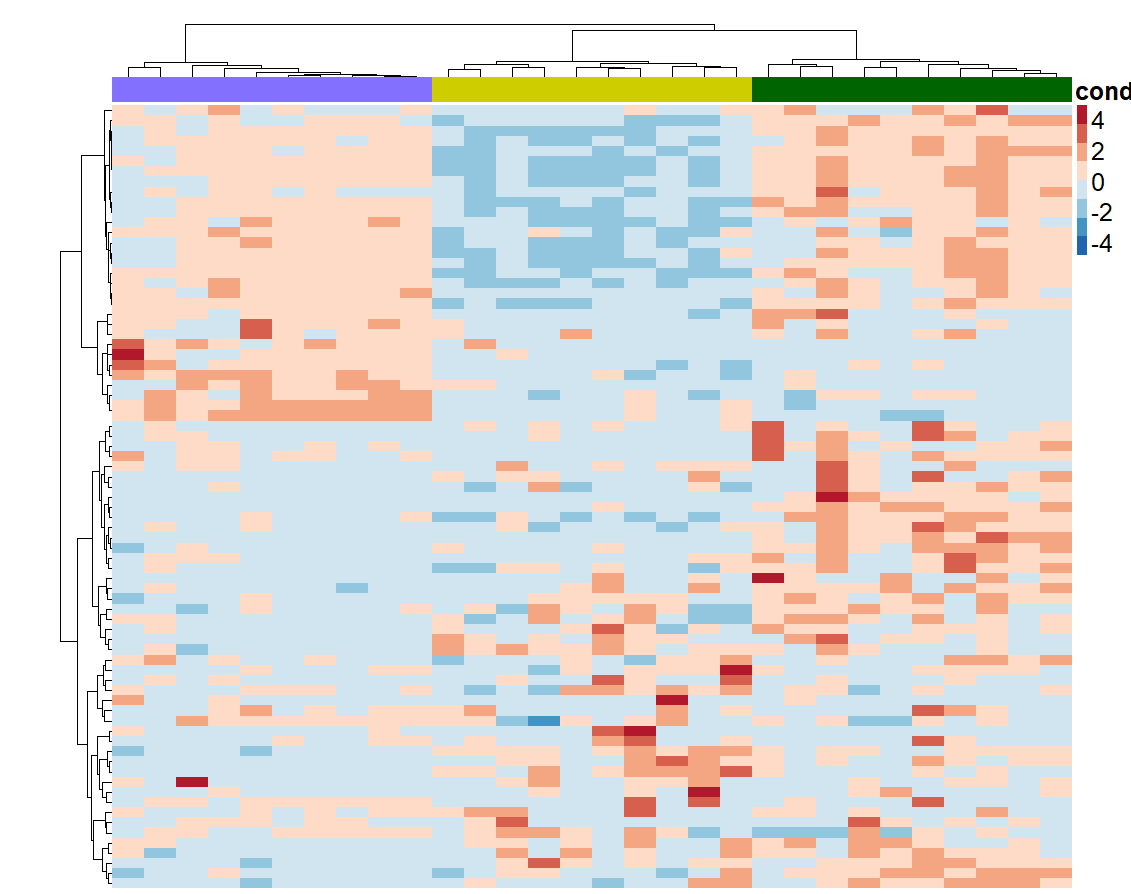

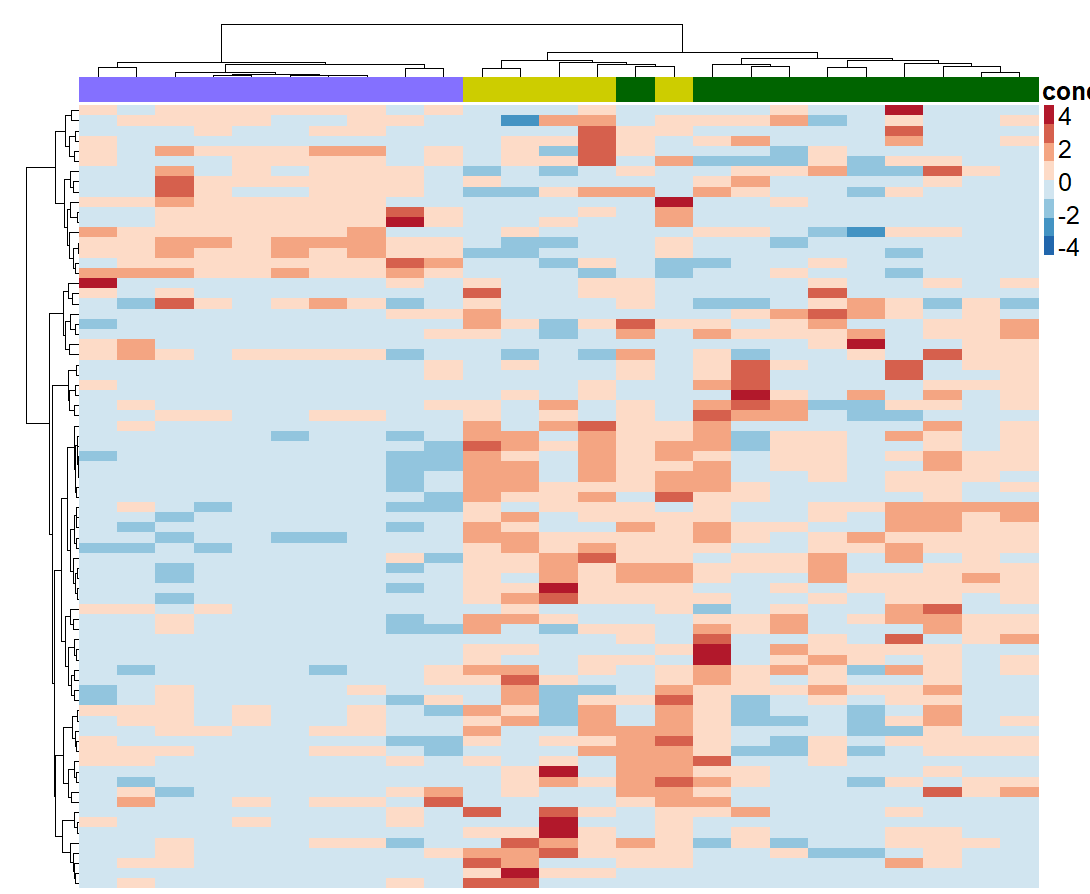

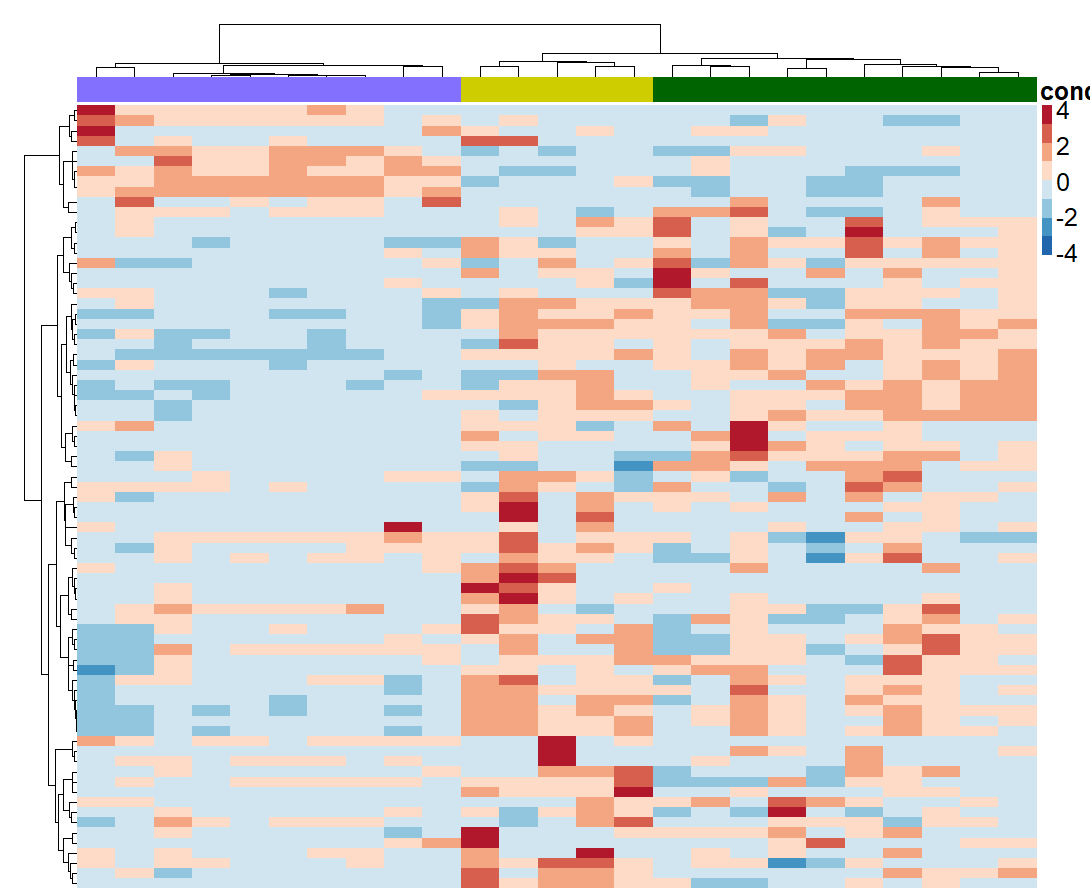


**Supplementary Figure 3: Transcriptomic profiling of compound treatment in 3D NASH hLiMTs confirms mechanism of action of clinical compounds, Firsocostat and Selonsertib, and reference compound, ALK5i.** **(a)** Principal component analysis of whole-transcriptome expression profiles from NASH, LEAN and compound-treated NASH hLiMTs on day 10. Distinct clusters for treatment groups suggest different mechanisms of actions for each compound. **(b-d)** Heatmaps of NASH, LEAN and compound treated NASH samples on day 10 for gene sets associated with hallmarks of NASH, namely adipogenesis (Hallmark, top row), inflammatory response (Hallmark, middle row) and collagen formation (Reactome, bottom row). Heatmaps are shown for treatment with 10 µM Firsocostat **(b)**, 2 µM Selonsertib **(c)** and 0.5 µM ALK5i **(d)**. In each heatmap, columns represent samples, and rows are genes. Row-wise Z-score transformation was performed on log2 expression values for each gene, with blue denoting a lower and red a higher expression level according to the average expression level. Data points are derived from single microtissues (n = 5-10).

**Supplementary Tables**

| code | gene_set |
| --- | --- |
| A1 | HALLMARK_TNFA_SIGNALING_VIA_NFKB |
| A2 | REACTOME_BIOLOGICAL_OXIDATIONS |
| A3 | WP_METAPATHWAY_BIOTRANSFORMATION_PHASE_I_AND_II |
| A4 | REACTOME_SIGNALING_BY_INTERLEUKINS |
| A5 | KEGG_DRUG_METABOLISM_CYTOCHROME_P450 |
| A6 | HALLMARK_INFLAMMATORY_RESPONSE |
| A7 | KEGG_METABOLISM_OF_XENOBIOTICS_BY_CYTOCHROME_P450 |
| A8 | HALLMARK_INTERFERON_GAMMA_RESPONSE |
| A9 | KEGG_RETINOL_METABOLISM |
| A10 | KEGG_CYTOKINE_CYTOKINE_RECEPTOR_INTERACTION |
| A11 | WP_IL18_SIGNALING_PATHWAY |
| A12 | REACTOME_PHASE_I_FUNCTIONALIZATION_OF_COMPOUNDS |
| A13 | WP_OXIDATION_BY_CYTOCHROME_P450 |
| A14 | KEGG_NOD_LIKE_RECEPTOR_SIGNALING_PATHWAY |
| A15 | KEGG_DRUG_METABOLISM_OTHER_ENZYMES |
| A16 | REACTOME_FATTY_ACID_METABOLISM |
| A17 | KEGG_CHEMOKINE_SIGNALING_PATHWAY |
| A18 | REACTOME_METABOLISM_OF_AMINO_ACIDS_AND_DERIVATIVES |
| A19 | HALLMARK_FATTY_ACID_METABOLISM |
| A20 | REACTOME_PHASE_II_CONJUGATION_OF_COMPOUNDS |
| A21 | REACTOME_INTERLEUKIN_10_SIGNALING |
| A22 | KEGG_STEROID_HORMONE_BIOSYNTHESIS |
| A23 | WP_TAMOXIFEN_METABOLISM |
| A24 | REACTOME_CLASS_A_1_RHODOPSIN_LIKE_RECEPTORS |
| A25 | HALLMARK_IL6_JAK_STAT3_SIGNALING |
| A26 | REACTOME_GPCR_LIGAND_BINDING |
| A27 | REACTOME_PEPTIDE_LIGAND_BINDING_RECEPTORS |
| A28 | WP_AMINO_ACID_METABOLISM |
| A29 | REACTOME_INTERLEUKIN_4_AND_INTERLEUKIN_13_SIGNALING |
| A30 | REACTOME_METABOLISM_OF_STEROIDS |

**Supplementary Table 1:** Enrichment map legend for GSEA comparing NASH versus LEAN at day 5 (Figure 5c). Legend shows full pathway names assigned to nodes.

| code | gene_set |
| --- | --- |
| B1 | HALLMARK_INTERFERON_GAMMA_RESPONSE |
| B2 | REACTOME_PEPTIDE_LIGAND_BINDING_RECEPTORS |
| B3 | WP_IL18_SIGNALING_PATHWAY |
| B4 | REACTOME_GPCR_LIGAND_BINDING |
| B5 | REACTOME_CLASS_A_1_RHODOPSIN_LIKE_RECEPTORS |
| B6 | HALLMARK_TNFA_SIGNALING_VIA_NFKB |
| B7 | KEGG_CYTOKINE_CYTOKINE_RECEPTOR_INTERACTION |
| B8 | REACTOME_SIGNALING_BY_INTERLEUKINS |
| B9 | REACTOME_CHEMOKINE_RECEPTORS_BIND_CHEMOKINES |
| B10 | HALLMARK_INFLAMMATORY_RESPONSE |
| B11 | REACTOME_INTERLEUKIN_10_SIGNALING |
| B12 | KEGG_CHEMOKINE_SIGNALING_PATHWAY |
| B13 | WP_SARSCOV2_INNATE_IMMUNITY_EVASION_AND_CELLSPECIFIC_IMMUNE_RESPONSE |
| B14 | WP_LUNG_FIBROSIS |
| B15 | HALLMARK_IL6_JAK_STAT3_SIGNALING |
| B16 | HALLMARK_INTERFERON_ALPHA_RESPONSE |
| B17 | WP_MIRNAS_INVOLVEMENT_IN_THE_IMMUNE_RESPONSE_IN_SEPSIS |
| B18 | REACTOME_SIGNALING_BY_GPCR |
| B19 | WP_FOLATE_METABOLISM |
| B20 | WP_PLATELETMEDIATED_INTERACTIONS_WITH_VASCULAR_AND_CIRCULATING_CELLS |
| B21 | REACTOME_REGULATION_OF_CHOLESTEROL_BIOSYNTHESIS_BY_SREBP_SREBF |
| B22 | HALLMARK_EPITHELIAL_MESENCHYMAL_TRANSITION |
| B23 | KEGG_NOD_LIKE_RECEPTOR_SIGNALING_PATHWAY |
| B24 | KEGG_RETINOL_METABOLISM |
| B25 | REACTOME_METABOLISM_OF_STEROIDS |
| B26 | REACTOME_INTERLEUKIN_4_AND_INTERLEUKIN_13_SIGNALING |
| B27 | WP_SELENIUM_MICRONUTRIENT_NETWORK |
| B28 | KEGG_STEROID_BIOSYNTHESIS |
| B29 | REACTOME_COMPLEMENT_CASCADE |
| B30 | WP_REGULATION_OF_TOLLLIKE_RECEPTOR_SIGNALING_PATHWAY |

**Supplementary Table 2:** Enrichment map legend for GSEA comparing NASH versus LEAN at day 10 (Figure 5d). Legend shows full pathway names assigned to nodes.

| code | gene_set |
| --- | --- |
| E1 | WP_METAPATHWAY_BIOTRANSFORMATION_PHASE_I_AND_II |
| E2 | HALLMARK_TNFA_SIGNALING_VIA_NFKB |
| E3 | KEGG_RETINOL_METABOLISM |
| E4 | KEGG_METABOLISM_OF_XENOBIOTICS_BY_CYTOCHROME_P450 |
| E5 | REACTOME_BIOLOGICAL_OXIDATIONS |
| E6 | KEGG_DRUG_METABOLISM_CYTOCHROME_P450 |
| E7 | REACTOME_PHASE_I_FUNCTIONALIZATION_OF_COMPOUNDS |
| E8 | WP_OXIDATION_BY_CYTOCHROME_P450 |
| E9 | REACTOME_XENOBIOTICS |
| E10 | REACTOME_CYTOCHROME_P450_ARRANGED_BY_SUBSTRATE_TYPE |
| E11 | KEGG_STEROID_HORMONE_BIOSYNTHESIS |
| E12 | WP_CONSTITUTIVE_ANDROSTANE_RECEPTOR_PATHWAY |
| E13 | REACTOME_METABOLISM_OF_STEROIDS |
| E14 | WP_TAMOXIFEN_METABOLISM |
| E15 | HALLMARK_INFLAMMATORY_RESPONSE |
| E16 | HALLMARK_INTERFERON_GAMMA_RESPONSE |
| E17 | WP_PREGNANE_X_RECEPTOR_PATHWAY |
| E18 | KEGG_DRUG_METABOLISM_OTHER_ENZYMES |
| E19 | WP_CHOLESTEROL_METABOLISM_INCLUDES_BOTH_BLOCH_AND_KANDUTSCHRUSSELL_PATHWAYS |
| E20 | HALLMARK_XENOBIOTIC_METABOLISM |
| E21 | HALLMARK_ALLOGRAFT_REJECTION |
| E22 | KEGG_CYTOKINE_CYTOKINE_RECEPTOR_INTERACTION |
| E23 | KEGG_LINOLEIC_ACID_METABOLISM |
| E24 | WP_NUCLEAR_RECEPTORS_IN_LIPID_METABOLISM_AND_TOXICITY |
| E25 | REACTOME_ACTIVATION_OF_GENE_EXPRESSION_BY_SREBF_SREBP |
| E26 | REACTOME_SRP_DEPENDENT_COTRANSLATIONAL_PROTEIN_TARGETING_TO_MEMBRANE |
| E27 | REACTOME_CHOLESTEROL_BIOSYNTHESIS |
| E28 | REACTOME_REGULATION_OF_CHOLESTEROL_BIOSYNTHESIS_BY_SREBP_SREBF |
| E29 | WP_CYTOPLASMIC_RIBOSOMAL_PROTEINS |
| E30 | REACTOME_SIGNALING_BY_INTERLEUKINS |

**Supplementary Table 3:** Enrichment map legend for GSEA comparing Firsocostat-treated samples to NASH samples at day 10 (Figure 6e). Legend shows full pathway names assigned to nodes.

| code | gene_set |
| --- | --- |
| D1 | HALLMARK_TNFA_SIGNALING_VIA_NFKB |
| D2 | WP_ALLOGRAFT_REJECTION |
| D3 | WP_SARSCOV2_INNATE_IMMUNITY_EVASION_AND_CELLSPECIFIC_IMMUNE_RESPONSE |
| D4 | REACTOME_PEPTIDE_LIGAND_BINDING_RECEPTORS |
| D5 | REACTOME_CLASS_A_1_RHODOPSIN_LIKE_RECEPTORS |
| D6 | HALLMARK_INFLAMMATORY_RESPONSE |
| D7 | REACTOME_GPCR_LIGAND_BINDING |
| D8 | HALLMARK_INTERFERON_GAMMA_RESPONSE |
| D9 | HALLMARK_IL6_JAK_STAT3_SIGNALING |
| D10 | KEGG_CYTOKINE_CYTOKINE_RECEPTOR_INTERACTION |
| D11 | REACTOME_INTERLEUKIN_10_SIGNALING |
| D12 | REACTOME_CHEMOKINE_RECEPTORS_BIND_CHEMOKINES |
| D13 | WP_MIRNAS_INVOLVEMENT_IN_THE_IMMUNE_RESPONSE_IN_SEPSIS |
| D14 | KEGG_TOLL_LIKE_RECEPTOR_SIGNALING_PATHWAY |
| D15 | WP_TOLLLIKE_RECEPTOR_SIGNALING_PATHWAY |
| D16 | REACTOME_SELENOAMINO_ACID_METABOLISM |
| D17 | WP_REGULATION_OF_TOLLLIKE_RECEPTOR_SIGNALING_PATHWAY |
| D18 | HALLMARK_IL2_STAT5_SIGNALING |
| D19 | REACTOME_SRP_DEPENDENT_COTRANSLATIONAL_PROTEIN_TARGETING_TO_MEMBRANE |
| D20 | REACTOME_SIGNALING_BY_INTERLEUKINS |
| D21 | KEGG_NOD_LIKE_RECEPTOR_SIGNALING_PATHWAY |
| D22 | PID_TCPTP_PATHWAY |
| D23 | REACTOME_METABOLISM_OF_AMINO_ACIDS_AND_DERIVATIVES |
| D24 | REACTOME_KERATINIZATION |
| D25 | REACTOME_FORMATION_OF_THE_CORNIFIED_ENVELOPE |
| D26 | HALLMARK_INTERFERON_ALPHA_RESPONSE |
| D27 | WP_IL18_SIGNALING_PATHWAY |
| D28 | WP_CYTOPLASMIC_RIBOSOMAL_PROTEINS |
| D29 | REACTOME_SIGNALING_BY_GPCR |
| D30 | REACTOME_NEGATIVE_REGULATION_OF_MET_ACTIVITY |

**Supplementary Table 4:** Enrichment map legend for GSEA comparing Selonsertib-treated samples to NASH samples at day 10 (Figure 7d). Legend shows full pathway names assigned to nodes.

| code | gene_set |
| --- | --- |
| C1 | HALLMARK_EPITHELIAL_MESENCHYMAL_TRANSITION |
| C2 | REACTOME_EXTRACELLULAR_MATRIX_ORGANIZATION |
| C3 | REACTOME_COLLAGEN_FORMATION |
| C4 | REACTOME_COLLAGEN_BIOSYNTHESIS_AND_MODIFYING_ENZYMES |
| C5 | KEGG_RETINOL_METABOLISM |
| C6 | REACTOME_ECM_PROTEOGLYCANS |
| C7 | REACTOME_ASSEMBLY_OF_COLLAGEN_FIBRILS_AND_OTHER_MULTIMERIC_STRUCTURES |
| C8 | KEGG_METABOLISM_OF_XENOBIOTICS_BY_CYTOCHROME_P450 |
| C9 | PID_AVB3_INTEGRIN_PATHWAY |
| C10 | REACTOME_COLLAGEN_CHAIN_TRIMERIZATION |
| C11 | KEGG_DRUG_METABOLISM_CYTOCHROME_P450 |
| C12 | REACTOME_COLLAGEN_DEGRADATION |
| C13 | REACTOME_ELASTIC_FIBRE_FORMATION |
| C14 | WP_METAPATHWAY_BIOTRANSFORMATION_PHASE_I_AND_II |
| C15 | REACTOME_DEGRADATION_OF_THE_EXTRACELLULAR_MATRIX |
| C16 | REACTOME_MOLECULES_ASSOCIATED_WITH_ELASTIC_FIBRES |
| C17 | HALLMARK_MYOGENESIS |
| C18 | REACTOME_BIOLOGICAL_OXIDATIONS |
| C19 | KEGG_DRUG_METABOLISM_OTHER_ENZYMES |
| C20 | REACTOME_XENOBIOTICS |
| C21 | PID_SYNDECAN_1_PATHWAY |
| C22 | KEGG_ECM_RECEPTOR_INTERACTION |
| C23 | KEGG_STEROID_HORMONE_BIOSYNTHESIS |
| C24 | KEGG_COMPLEMENT_AND_COAGULATION_CASCADES |
| C25 | PID_INTEGRIN1_PATHWAY |
| C26 | WP_OXIDATION_BY_CYTOCHROME_P450 |
| C27 | REACTOME_NON_INTEGRIN_MEMBRANE_ECM_INTERACTIONS |
| C28 | KEGG_STARCH_AND_SUCROSE_METABOLISM |
| C29 | HALLMARK_XENOBIOTIC_METABOLISM |
| C30 | REACTOME_SYNDECAN_INTERACTIONS |

**Supplementary Table 5:** Enrichment map legend for GSEA comparing ALK5i-treated samples to NASH samples at day 10 (Figure 8c). Legend shows full pathway names assigned to nodes.
